# Physiological response and secondary metabolites of three lavender genotypes under water deficit

**DOI:** 10.1038/s41598-021-98750-x

**Published:** 2021-09-27

**Authors:** Hossein Gorgini Shabankareh, Sarah Khorasaninejad, Hasan Soltanloo, Vahid Shariati

**Affiliations:** 1 Horticultural Sciences Department, Gorgan University of Agricultural Sciences and Natural Resources, Gorgān, Iran; 2 Plant Breeding Department, Gorgan University of Agricultural Sciences and Natural Resources, Gorgān, Iran; 3 National Institute of Genetic Engineering and Biotechnology, Tehran, Iran

**Keywords:** Developmental biology, Ecology, Physiology, Plant sciences, Systems biology

## Abstract

*Lavandula* genus is a considerable medicinal plant in pharmaceutical and cosmetics industries. Considering increasing threat of drought in the world, it is important to identify genotypes which can tolerate drought. It is also important to characterize quantity and quality of essential oils, and tolerance indicators of these genotypes against drought stress. Therefore, an experiment was conducted in Gorgan University of Agricultural Sciences and Natural Resources, Iran, during 2017 and 2018, to investigate these factors. It was a factorial experiment based on randomized complete block design with two treatments, three genotypes (*Lavandula angustifolia* cv. Hidcote, *Lavandula angustifolia* cv. Munstead, and *Lavandula stricta*), and four levels of drought stress (irrigation regimes) (I_1_: 100–90% (control), I_2_: 80–70%, I_3_: 60–50% and I_4_: 30–40% of field capacity) which was done with three repetitions. Drought increased amount of proline in leaves, antioxidant activity, activity of catalase, peroxidase, ascorbate peroxidase, and superoxide enzymes, malondialdehyde content, total flavonoids, total phenol, total sugar and essential oil percentage. The PCA analysis of different irrigation regimes showed that in the first component, the best traits are antioxidant enzymes CAT, SOD, APX, while in the second component, only the trait Catalase is the best trait. The results of PCA analysis in lavender genotypes showed that *L. stricta* exhibits the most affected physiological changes while trying to adjust to changes in the water status of the environment, under the imposed conditions and shows the highest resistance. But it reduced dry weight of aerial parts, relative water content of leaves, and efficacy of essential oil. *Lavandula stricta* genotype had the highest amount of essential oil, but the highest dry weight of the aerial parts and essential oil yield were related to *L. angustifolia* cv. Hidcote and *L. angustifolia* cv. Munstead genotypes. In all evaluated genotypes, with increasing drought stress, monoterpene compounds were decreased and sesquiterpene compounds were increased. Totally it was shown that drought effect on evaluated traits depends on genotype and nature of traits; this indicates that by choosing drought-tolerant genotypes in breeding programs, high quantity and quality of essential oil, as well as tolerance to drought stress can be achieved.

## Introduction

Global warming brings several problems, including the raising of drought stress worldwide. Climate change is expected to increase the frequency of water deficit, heat stress and increased saline soils. Drought stress is one of the most important abiotic stress which causes a significant decrease in the growth and yield of most plants^1^. It damages the electron transport chain, leading to the producing of reactive oxygen species (ROS)^2^. ROS such as O_2_^−^, H_2_O_2_, and OH^−^ may collect during drought stress and harm the photosynthetic system and ruin normal metabolism via oxidative damage of proteins, lipids and nucleic^3^. The protection against oxidative damages is provided by both non-enzymatic and enzymatic antioxidants in the chloroplasts of the plant cells^4^. Antioxidant enzymes including superoxide dismutase (SOD), catalase (CAT), and ascorbate peroxidase (APX) decline the injurious effects of ROS on plant cells and defend them against oxidative damage^5^.

Furthermore, the secondary metabolites accumulation such as essential oils (EO) is considered as a protection mechanism by the plants to moderate to these stresses^6^. Increasing in yield and changing in composition of essential oil under drought stress has been reported in two *Salvia* species and Sweet Basil^7,8^. Other studies have emphasized that volatile organic compounds (VOCs) biosynthesis is mostly influenced by multiple biotic and abiotic agents and researchers have focused to modified EO quality and quantity through the studies of the molecular mechanisms involved in plant-environment interactions^4, 9^.

Recently, the medicinal plants were cultivated mostly in a water deficit stress condition to affect the secondary metabolites content, like alkaloids and phenols^10^. The essential oil of medicinal plants are one of the most commercially important agricultural products. These products are widely used in drinks, flavoring, cosmetics perfumeries and pharmaceuticals industries^11^. The essential oils production rate was 40–60 thousand tons with 700 million dollars profitability in 2012 and according to the present analysis of data and reports, the global market of essential oils (EO) was valued at USD 7.6 Billion in 2018 and is envisaged to reach USD 15.1 Billion by the year 2026, at a CAGR of 8.7% shows that the use of these products is growing around the world^12,13^.

*Lavandula* spp. are perennial herb cultivated basically for its flowers and flowering tops which produces valuable essential oil and are favorite since old times and has been used widely either dried or as an essential oil in aroma therapy, as a pesticide and antimicrobial agent^14^. The major economic importance of lavender is due to its high essential oil (EO) quality, of which 200 tons are produced every year globaly^15^. The lavender EO is considered one of the most medically beneficial EOs. It has antifungal and antibacterial activities and is being used to neurological disorders and treat infections^15,16^. The flower oil contains mainly of linalool, linalyl acetate, camphor and 1, 8-cineole, while oil originating from leaves contains mainly borneol, camphor and 1, 8 cineole^14,17^. The most important lavender in the world are *Lavandula angustifolia* and Lavendin (*L. angustifolia* × *L. latifolia*) that commercially cultivated all over the world^15^. Therefore, many breeding projects have been done on these species, especially English lavender, and various varieties have been introduced, the most well-known cultivars are *Lavandula angustifolia* ‘Hidcote’ and *Lavandula angustifolia* ‘Munstead’^18,19^.

Also, in the flora of Iran, the genus *Lavandula* is represented by two species, *Lavandula stricta* Del. and *Lavandula sublepidota* Rech.^20, 21^. *Lavandula stricta* (Syn. *L. coronopifolia* Poiert) is a wood-based perennial forming a large bush from the Cape Verde Islands, across North Africa, West Tropical Africa, NE Tropical Africa, Western Asia (Jordan and Iran) and the Arabian Peninsula that is found in open rocky and stony habitats and desert plains from sea level to ca. 2000 m ^22–26^. The dried leaves, flowers, and stems of *L. stricta* are traditionally used as stomachic and appetite stimulants while being exported to the countries on the margin of the Persian Gulf and Oman Sea^27,28^. Since this spices is native to arid and semi-arid regions, it is expected to have a lower water requirement than commercial lavender species such as *L. angustifolia* and has a high potential for tolerating drought conditions.

In addition, it is clear that in the arid and semi-arid areas, water deficiency is an important stress reducing the plants productivity, leading to the decline of photosynthesis and hence, the plant growth. Since drought can have a considerable impact on the plant entire metabolism, the secondary metabolites production is also affected by it^27^. So, there is a need to find new variation sources to afford with these stresses and for cultivating, breeding new genotype (hybridization between two species, or even though transgenic plants). Efficient and effective screening methods must be developed to identify and dissect the physiological basis of tolerance to abiotic stresses. Therefore, the aim of the current work was to compare the impact of drought stress among *Lavendula angustifolia* cv. Hidcote, *Lavendula angustifolia* cv. Munstead and *Lavendula stricta* with emphasizing on the yield and chemical composition of essential oils through GC/MS analysis. Moreover the antioxidant activity as well as the quantification of phenols and flavonoids where determined.

## Materials and methods

### Plant material

The seeds of *L. angustifolia* cv. Hidecot and *L. angustifolia* cv. Muneasted were obtained from Renees Garden USA. In order to break the seed dormancy, the seeds were placed in May 2017 in a completely humid medium for 3 weeks at 4 °C and in June 2017, the seeds were planted in the seedling tray.

Also, the seeds of the *L. stricta* were collected at the maturity stage from their natural habitat, the Geno protected area, Bandar Abbas, Hormozgan province, southern Iran (latitude 27° 18′ to 27° 29, longitude 56° 18′ to 56° 55′; altitude 200 m from sea level). So that the mother plants of *Lavandula stricta* were identified by the expert of the Research and Training Center for Agriculture and Natural Resources of Hormozgan Province (Bandar Abbas—Iran) and with the permission of this center, their seeds were collected and transferred to the Agricultural Research and Training Center and Natural Resources of Golestan Province (Gorgan-Iran) along with the herbarium sample (Under letter No. 6452/283/11). Voucher specimens were deposited at the Herbarium of Golestan Agricultural and Natural Resources Research and Education Center, Gorgan, Iran (Voucher No.: 4519). In a study conducted by Sanginabadi et al. (2016) was found that such seeds do not require chilling for germination which is reasonable given the plant's habitat and requirements^29^.

About 3 months after the planting in the seedling tray (September 2017) to prepare the seedling, one plant was planted inside each pot (pots of 6 cm diameter and 10 cm height). After about 4 months, in January 2018, the seedlings were transferred to the main pots (plastic pots with the opening diameter of 30 cm and height of 40 cm) in the ten-leaf stage. A total of 108 pots (including the subunits in each replication) were used for the planting. After 3 months, once the seedlings were adapted, the pots were moved to the open space in the mid-April 2018. After the plant growing, the weed was manually removed in the experimental units three times (one stage before irrigation treatments, second stage in mid-plant growth period and third stage before flowering period).

### Experimental design

The present research was done in Gorgan University of Agricultural Sciences (latitude 36°30 N; longitude 53°57 E; 155 m altitude) in 2017–2018. In the first year, the perennial plants were established, whereas parameters were measured in the 2nd year. A factorial experiment (two treatments) was performed in the randomized complete block design with three replications. Treatments were three genotypes (*Lavandula gngustifolia* cv. Hidecot, *Lavandula angustifolia* cv. Muneasted and *Lavandula stricta*) and four levels of drought stress (Irrigation Regime) as follows (I_1_: 90–100% field capacity (FC), I_2_: 70–80% FC, I_3_: 50–60% FC and I_4_: 30–40% FC).

### Drought treatments

Drought stress treatment was done through the weighted method. The pots were consistently irrigated 20 days following planting. The samples were exposed to drought stress, whereas the control samples were grown in pots with the similar status (30 cm in diameter × 40 cm in depth). The standard garden soil (8 kg to each pot using scales) was added to the pots. Then the soil was saturated by adding water to the pots. They were located on the grid surface (48 h) to let excess water drain out for reaching to the field capacity (FC)^30^. Then soil moisture percentage (%) was assessed in the FC of the farm. To perform various moisture treatments, the soil moisture was measured to let water deficit calculation by weighing the sample pot in each block. In the next step, the pots were added with the required water. For controlling the samples’ dry weight, one additional pot was considered for the moisture treatments (totally four pots for index) and was used for adding their dry weights to the weights obtained from the pots as well as for allocating an appropriate amount of water to the pots while moisture treatments. Index pots were weight every day and moisture deficiency were calculated for assessing the needed level of irrigation to perform drought stress treatments. The regimes, by which the moisture weight percentage of the soil water content was close to the drought condition (filed capacity percentage), were considered in treatments^31,32^.

### Leaf relative water content

The whole leaves were used for measuring the RWC, as the leaves of same size were isolated at the time of complete vegetative growth and their fresh weight was measured by accurate laboratory scale. The leaves were then immersed in the closed petri dishes in distilled water and placed at low light intensity for 15 h. They were placed between two layers of Whitman No. 1 filter paper for about 1 min to be dried and their turgid weight was immediately measured. Then, the leaf parts were dried in oven at 70 °C for 48 h and their dry weight was determined and the RWC of treatments was measured using Eq. (1) ^33^.

1 $$\% {\text{RWC}} = [({\text{Wf}}{-}{\text{Wd}})/({\text{Wt}}{-}{\text{Wd}})] \times 100$$

In this equation, Wf represents the leaves fresh weight, whereas leaf turgid weight is shown by Wt and Wd indicates the leaf dry weight.

### Measurement of proline content

For measurement of proline concentration method of Bates et al. was used^34^. According to this method 0.2 g leaves were placed in 10 ml 3% sulfosalicylic acid aqueous solution and it remained still for 24–48 h, after filtering the resulting solution, 2 ml of this solution was mixed with 2 ml ninhydrin reagent and then 2 ml acetic acid was added to each tube. Sample were placed in 100˚C for 1 h, in a water bath, and then placed on ice. In next step to each tube 4 ml toluene was added to each tube and samples were homogenized using a mixer. The absorption of resulting upper colored phase of samples was measured using a spectrophotometer device at 520 nm wave length. Using proline standard curve, proline concentration in each treatment was determined.

### Soluble sugar

An amount equal to 0.1 g dried leaves were completely powdered, and 13 ml of 80% ethanol was added to each sample in a centrifuge tube; and then each tube was centrifuged for 10 min at 500 rpm. The supernatant was separated and then 10–12 ml 80% ethanol was added to the resulted pellet; and centrifuged for 10 min in 5000 rpm. Again the supernatant was separated and was added to last step’s supernatant, this supernatant was the essential oil. After that, 1 ml of the essential oil was poured in to a test tube and 1 ml 5% phenol was added to each tube. Then immediately 5 ml of concentrated sulfuric acid was added to each tube. It was shaken, placed in oven for 30 min, and then placed in cold water. Absorption of samples was measured in 490 nm wave length using a spectrophotometer. Glucose solutions with different concentrations were used to draw a standard curve (in mg/g of dry weight)^35^. In next step the formula was calculated using the standard curve, and spectrophotometer number 485 nm absorption was placed in the formula and sugar content was calculated using Eq. 2.

2 $${\text{Sugar}}\;{\text{mg}}\;{\text{g}}^{{ - {1}}} {\text{ dw}} = \left( {{\text{X}}*104.84} \right) - 3.28$$

### Shoot fresh and dry mass

Aerial parts of the samples were collected at the final flowering phase from three middle lines (35 cm from each side). For shoot dry mass determination, the specimens were dried in an oven (48 h/70 °C). Therefore, the total shoot dry matter in each hectare was assumed for plots (kg/hectare)^31,32^.

### Determination of total phenolic content

To evaluate phenolic compound, first methanolic extract was prepared. To prepare methanolic extract, the samples were dried in shadow, then were powdered using an electric mixer. Of each sample 1 g was dissolved in 10 ml 80% methanol. The samples were incubated for 24 h on a shaker. Afterwards, each sample was filtered separately using filter paper, and was centrifuged for 4 min at 3100 rpm ^36^.

To measure total phenol, 20 µl of methanolic extract (1 g in 10 ml methanol) was mixed with 100 µl Folin–Ciocalteu reagent (FCR) and 1.16 ml water. After 5–8 min, 300 µl of 1 M sodium carbonate (10.6 g in 100 ml water) was added to each sample. This solution was placed in dark and 40˚C water-bath for 30 min. For control, instead of extract, 80% methanol was used. The control solution was used to calibrate the spectrophotometer device (UVVRS, 2008, USA). Absorption of samples were measured in 765 nm wave length. To draw the standard calibration curve, different concentrations of gallic acid, a solution of gallic acid in methanol: water (50:50) were used (Eq. 3) ^37^.

3 $${\text{Y}} = 0/0{\text{166x}}{-}0/{1622}$$

Y = the absorption of sample in 760 nm.

### Determination of total flavonoid content

To measure flavonoid content, the method of Chang et al. was used (2012). First, 0.5 ml of prepared methanolic extract was mixed with 1.5 ml methanol, 0.1 ml of 10% aluminum chloride in ethanol (10 g aluminum chloride in 100 ml ethanol and distilled water), 0.1 ml of 1 M potassium acetate (2.41 g in 10 ml distilled water) and 2.8 ml distilled water. Pure methanol was used as control, instead of methanolic extract. This solution was placed in dark for 30 min and immediately afterwards, the absorption was measured in 415 nm. The total flavonoid content was determined using quercetin standard curve. To do so, different concentrations of quercetin standards were used to achieve a linier equation, with which the flavonoid content (1n 100 g of dry material) was calculated^38^.

### Antioxidant activity evaluation-DPPH assay

To measure inhibition of free radicals (antioxidant activity) of samples, DPPH (1, 1-diphenyl-2-picrylhydrazyl) was used. First, 1 ml of methanolic extract was mixed with 1 ml of 0.1 mM DPPH. As control sample instead of methanolic extract, 1 ml pure methanol was added. Pure methanol was used as blank. The samples were placed in dark for 30 min and then the absorption was measured in 517 nm wave length by means of a spectrophotometer device. Resulted numbers were transformed in to percentage of inhibition of free radicals using Eq. 4^39^.

4 $${\text{Inhibition}}\;{\text{percentage}}\;\left( {\% {\text{IP}}} \right) = \left[ {{1} - \left( {{\text{As}}/{\text{Ac}}} \right)} \right] \times {1}00$$

In this Equation, Ac represents control absorbance, and As shows the tested sample absorbance.

The resulted numbers are inhibition percentage of free radicals in methanolic extract of samples (ppm1/0).

### Evaluation of enzymatic activity of antioxidant enzymes

#### Drawing out extract to measure enzymatic activity

An amount equal to o.5 g fresh leaves were smashed using liquid nitrogen in a crucible, then 1 ml of 50 mM phosphate buffer containing 0.5 M EDTA and 2% PVPP was added to the smashed tissue. It was centrifuged for 20 min, at 14,000 rpm and 4˚C. The supernatant was used to measure enzymatic activity of catalase, peroxidase, ascorbate peroxidase, and superoxide dismutase enzymes^40^.

#### Peroxidase enzyme activity

To measure enzymatic activity of peroxidase, 33 µl of the extract was mixed with 1 ml peroxidase solution containing 13 mM guaiacol, 5 mM H_2_o_2_, and 50 mM potassium phosphate buffer (pH = 7). The absorption was measured in 1 min, with 10 S intervals, at 470 nm wave length (in terms of µmolar H_2_o_2_ consumption per minute). To make 100 ml potassium phosphate buffer 39 ml of 50 mM monobasic potassium phosphate was mixed with 61 ml of 50 mM dibasic potassium phosphate^41^.

#### Ascorbate peroxidase enzyme activity

To measure activity of this enzyme, 50 µl of extract, was mixed with 1 ml of ascorbate peroxidase measurement solution. This solution contains 50 mM potassium phosphate buffer (pH = 7), 0.1 mM EDTA, 0.5 mM ascorbic acid, and 0.15 mM H_2_O_2_. After 1 min, absorption of the sample was measured at 290 nm wave length using a spectrophotometer device^42^.

#### Catalase enzyme activity

To measure catalase enzyme activity, 50 µl of the extract was mixed with 1 ml catalase measurement solution, which was consisted of 50 mM potassium phosphate buffer (pH = 7) and 15 mM H_2_O_2_. After 1 min, absorption of sample was measured at 240 nm using a spectrophotometer device (in terms of µmolar H2o2 consumption per minute)^43^.

#### Superoxide dismutase enzyme activity

To measure activity of this enzyme, 50 µl of the extract, was mixed with 1 ml of SOD measurement solution containing 50 mM potassium phosphate buffer (pH = 7.8), 75 µM NBT (Nitro Blue Tetrazolium), 13 mM L-methionine, 0.1 mM EDTA, and 2 µM riboflavin. This solution was placed in light-room for 15 min. Then absorption was measured at 560 nm wave length (in terms of µmol per g)^43^.

#### Malondialdehyde in leaves

To determine malondialdehyde concentration in leaves, first 0.5 g fresh leaves were powdered completely in a solution containing 20% TCA (three chloric acid) and 30% thiobarbituric acid. This mixture was placed in 95˚C in water-bath for 25 min. Then it was cooled in ice-bath; and the concentration of malondialdehyde was measured at 532 nm wave length using the method of Valentinovich et al. (2006)^44^.

### Essential oil components

#### Essential oil distillation

Aerial parts of the samples were collected to assess shoot dry matter at the flowering period. Aerial parts of the plants (flowering tops) were dried in shade and room temperature and grounded in a grinder with three replications. The air-dried plant materials were grounded and were steam distilled for 3 h in a Clevenger-type apparatus, where vapor from heated water crosses the plant material carrying the volatile compounds to a condenser tube^45^. The obtained oils were dried over anhydrous sodium sulfate and stored in a dark glass bottle at 4 °C until analysis. The oils were yellowish in color. The essential oil percentage (%, v/ w) and yield (g m − 2) were calculated according to Eqs. (5) and (6), respectively^45^ with sight modifications:

5 $${\text{Essential oil content \% }} = \left( {\frac{{{\text{Extracted essential oil }}\left( {{\text{mL}}} \right)}}{{50{\text{ g of dried plant ground sample}}}}} \right) \times 100$$

6 $${\text{Essential}}\;{\text{oil}}\;{\text{yield}}\;{\text{g}}/{\text{plant}} = {\text{Lavender}}\;{\text{biomass}}\;{\text{yield}}\;{\text{g}}/{\text{plant}} \times {\text{Essential}}\;{\text{oil}}\;{\text{content }}\%$$

### Essential oil analysis

#### Gas chromatographic-mass spectrometric

Analysis was performed using a TRACE MS instrument (Thermo Quest-Finnigan) equipped with a DB-5 column (30 m × 0.25 mm, film thickness 0.25 μm). The flow-rate of helium carrier gas was 1.1 mL/min, final temperature 200 °C while detector temperature was set at 250 °C; MS spectra were taken at 70 eV (E1) over the range 40–460 amu with an electron multiplier voltage of 1800 eV and scan time was 0.4 scans/ sec. The constituents of the essential oils were identified by calculating retention indices under temperature-programmed conditions for n-alkanes (C6–C20), and the oil on a DB-5 column under the same conditions. Individual compounds were identified by comparing their mass spectra with those of an internal reference mass spectra library (Wiley 7.0) or of authentic compounds, and confirmed by comparing their retention indices with those of the authentic compounds or those reported in the literature^46^. Semi-quantitative data was obtained from FID area percentages without using correction factors. The analysis was carried in Phytochemistry Laboratory of Medicinal Plants and Drugs Research Institute, Shahid Beheshti University, Tehran, Iran.

#### Soil and weather data

**Figure 1:**
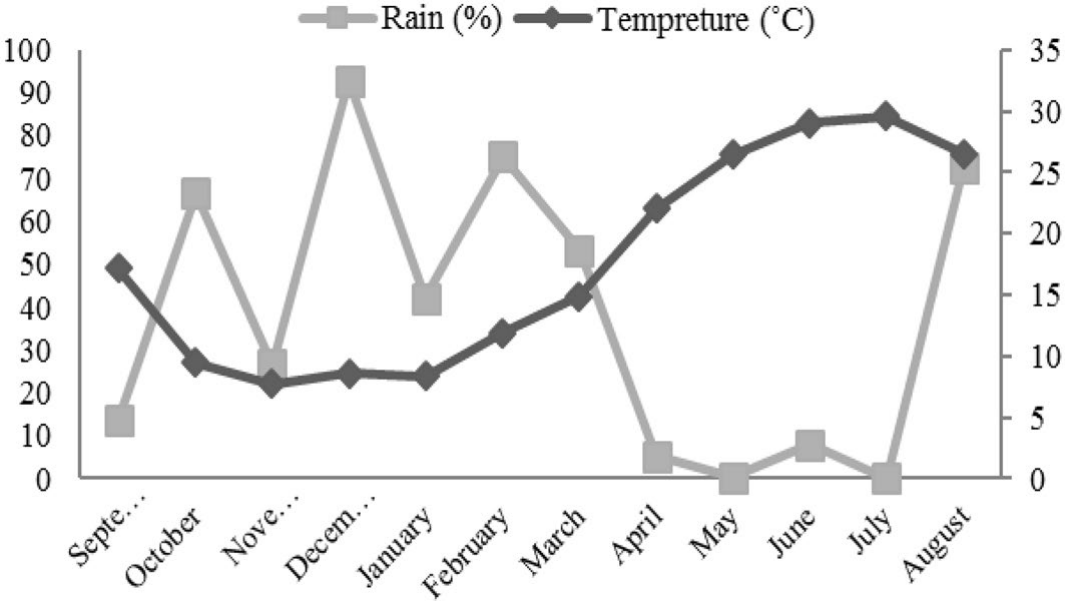
Meteorological data from September 2017 to August 2018 for the experimental site of the study (National Meteorological organization, Iran).

In order to determine the quantitative and qualitative characteristics of soil, a soil sample was taken to the laboratory and the chemical and physical analyses were carried out (Table 1). The seasonal pattern of climatic condition from September 2017 to August 2018 has been illustrated in Fig. 1.

**Table 1:** The soil physical and chemical analysis.

Lab. No^1^	pH^2^	EC*10^3^	SP^4^	TNV^5^	N^6^%	OC^7^%	P(ava)^8^ ppm	K(ava)^9^ ppm^10^	%Clay	%Silt	%Sand	texture
947	7.4	4.076	141.86	5.59	0.09	0.9	24.8	256	12	42	46	Loam

1. Laboratory Number, 2. Power of Hydrogen, 3. Electrical Conductivity, 4. Saturation Percentage, 5. Total Neutralizing Value, 6. Nitrogen, 7. Organic carbon, 8. Phosphorus, 9. Potassium, 10, part per million.

#### Data analysis

SAS 9.1 was used for data analysis and comparison of the obtained treatments averages was performed by the least significant difference (LSD) tests (*p* < 0.05), CIM miner-One Matrix software was used to draw the Heat map diagram for the essential oil profile in lavender genotypes corresponding to the different irrigation regimes. The physiological and biochemical traits for the classification cultivars in response to drought stress were subjected to Principal Component Analysis (PCA) using R 4.0.4 packages.

The present study complies with relevant institutional, national, and international guidelines and legislation.

## Results and discussion

### Wet and dry weight of aerial parts

**Figure 2:**
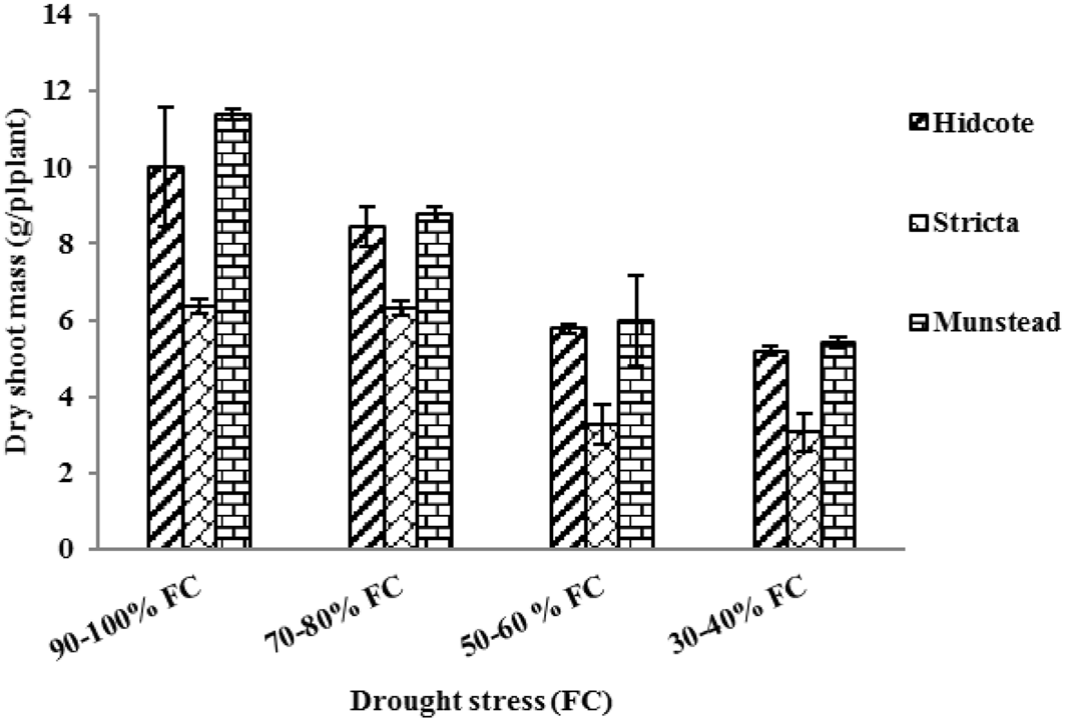
The effect of drought stress on dry weight of aerial parts in different lavender genotypes.

Dry weight of aerial parts was significantly affected by drought stress and genotype treatments and their interactions (Table 2). With increasing drought stress the amount of dry weight of aerial parts in all genotypes was decreased. Dry functions in I_2_, I_3_, and I_4_ levels in H genotype (*Lavandula gngustifolia* cv. Hidecot) were 15.68%, 40.35% and 48.15%, respectively. In S genotype (*Lavandula stricta)* these amounts were 0.78%, 48.58% and 51.72%, respectively; and in M genotype (*Lavandula angustifolia* cv. Muneasted) they were 22.29%, 49.38% and 52.63%, respectively. Compared to the control group, the most reduction in dry weight of aerial parts was in M genotype. The highest amount of dry weight (11.40 g in plant) was observed in H genotype in drought stress of 90–100% of field capacity. The lowest amount of dry weight of aerial parts (3.07 g) was seen in S genotype in drought stress of 30–40% field capacity (Fig. 2).

**Table 2:** Variance analysis of the effect of drought stress on enzymatic activity of antioxidant enzymes, and quantity of essential oil from different lavender genotypes.

Mean of squares						
Traits	Replications (R)	Genotype (G)	Drought (I)	Genotype × Drought (GI)	Error	CV (%) (coefficient of variation)
df (degree of freedom)	2	2	3	6	22	
Shoot fresh mass	4.13^ns^	695.93**	867.16**	19.28*	7.41	8.83
Shoot dry mass	0.50^ns^	33.47**	46.01**	1.33*	0.39	9.38
Leaf relative content	66.72^ns^	1291.59**	3259.65**	55.24*	17.16	8.34
Leaf proline content	0.069^ns^	7.57**	10.21**	0.30**	0.017	4.50
Antioxidant activity	31.31^ns^	249.69**	1688.86**	275.96**	15.55	8.46
Soluble sugar	14.35^ns^	242.50**	1273.67**	35.76^ns^	14.66	9.33
Total phenol	0.007^ns^	0.26**	0.58**	0.095**	0.018	7.52
Flavonoid	0.008^ns^	0.114**	0.033**	0.0002^ns^	0.0037	6.08
SOD^1^	1800.30^ns^	8962.44**	20,217.54**	2221.57*	633.96	10.69
POX^2^	363.15^ns^	4441.35**	14,216.52**	1111.11*	308.88	8.05
APX^3^	6058.62^ns^	24,763.46**	20,851.38**	164.69^ns^	1531.07	16.64
CAT^4^	1750.33^ns^	3301.10**	54,584.62**	8085.51**	493.20	6.40
MDA^5^	2.90^ns^	22.45**	61.41**	4.40*	1.48	15.68
Essential oil rate (%)	0.032^ns^	0.348**	1.17**	0.194**	0.013	14.32
EO^6^ yield g/plant	0.00028^ns^	0.00031*	0.0013**	0.00026**	0.000062	16.92

1. SOD, Superoxide dismutase; 2. POX, Peroxidase; 3. APX, Ascorbate peroxidase; 4. CAT, Catalase; 5. MDA, Malondialdehyde; 6. EO, essential oil.

^ns^No significant difference.

*Significant difference at *p* < 0.5.

**Significant difference at *p* < 0.1.

In this study drought stress had a negative effect on biomass of lavender plants. This effect can be due to water shortage. Because drought stress cause reduction in swelling, total water potential in cell and withering, it also results in closing stomata, reduction in cell division, and cell enlargement^47,48^. Reduction in cell division and cell enlargement as a result of drought, reduce the leaf surface, photosynthesis and growth function of the plant. In other words, reduction in photosynthesis products, cause reduction in leaf’s surface; and reduction in transfer of assimilated materials to aerial part, as a result of drought, cause decrease in aerial yield of the plant^49^. In this regard, Abbaszadeh et al. (2020) reported that due to drought stress of 30% and 60% of field capacity, dry weight of aerial parts in *Rosmarinus officinalis* L. has decreased. While contrary to our results Rhizopoulou and Diamantoglou (1991) observed that dry weight of leaves from Marjoram plant (*Origanum majorana)* was increased with increased soil moisture deficiency; which can be due to differences in plant species and ecological conditions^50,51^.

### Proline content of leaves

**Figure 3:**
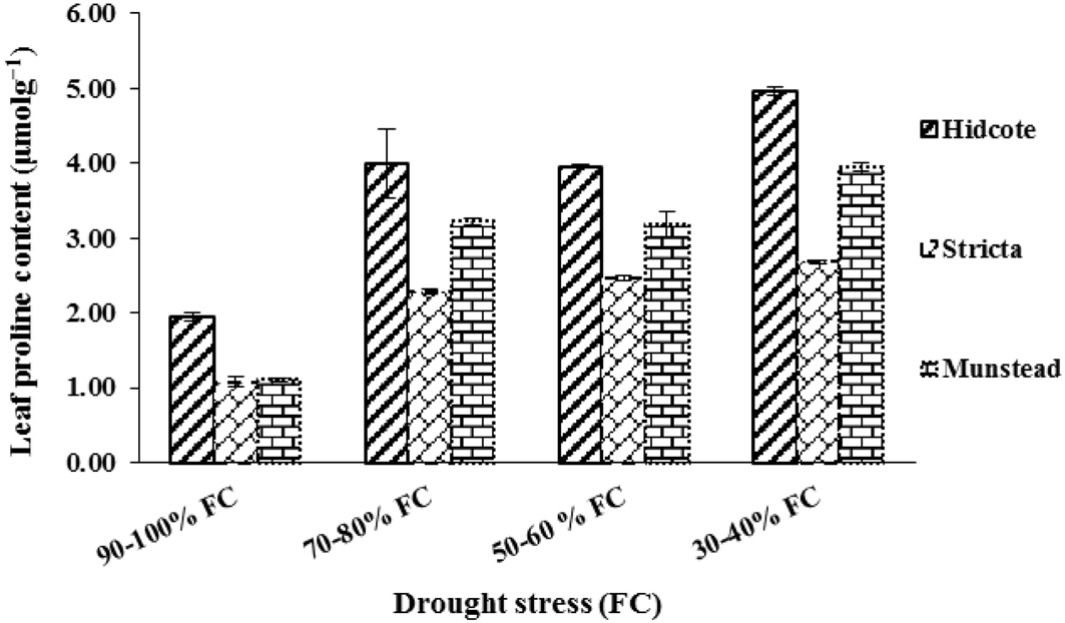
The effect of drought stress on proline content in different genotypes of lavender.

The results of variance analysis showed that drought stress, genotype and their interactions have significantly affected proline content of leaves (Table 2). With increasing drought stress the proline content was increased. The highest amount of proline content (4.96 mg per g) was observed in H genotype in I_4_ drought level (30–40% of field capacity). While the lowest amount (1.08 mg per g) was observed in S genotype in irrigation of 90–100% of field capacity (Fig. 3). In each genotype separately, in I_2_ to I_3_ drought levels the amount of proline was equal, but in H and M genotypes with increasing drought stress, the amount of proline was increased, While in S genotype with increasing water deficit proline did not show a significant increase. This indicates that two genotypes (H and M) have a similar function for using these types of osmolyte to deal with this level of drought. Which this result may be exist another osmolite production as a resistance mechanism in S genotype^52^.

One change that happens in biological and non-biological stresses is increasing the amount of osmolytes in plant. To prevent negative effects of drought stress, the plant increases the amount of its osmolytes including proline^53^. Proline is an amino acid which in addition to act as an osmolyte, plays an important role in maintaining and stabilizing membranes by adding membrane phospholipids and changing the hydrated layer around macromolecules. Proline is also recognized as a stabilizer for cellular homeostasis under stressful conditions. This is due to high ability of proline to stabilize sub-cellular structures such as proteins and cell membranes and its ability to eliminate free radicals^54^. In present study, increasing proline content in different genotypes of lavender as a result of drought, can be for the same reason. It is proved that in some plants, changes in amount of proline is related to their ability to tolerate and adapt with drought stress; so, the proline content can be used as an indicator to select drought-resistant plants. Hosseinpour et al. (2020), reported that in response to drought stress, accumulation of compatible metabolites such as proline can participate in water absorption. In accordance with our results, an increase in proline content in different genotypes of *Calendula officinalis* plant due to drought stress has been reported as well^55,56^. However, in some plant species, other osmolites are produced under biological stress, the most important of them is glycine betaine. So that it is probable that the relationship between glycine betaine accumulation and stress tolerance, such as drought stress, is species- or even genotype specific^57^. As a results, the S genotype likely produced glycine betaine under drought stress, obviously, completed studies are needed to confirm this hypothesis.

### Relative water content of leaves

**Figure 4:**
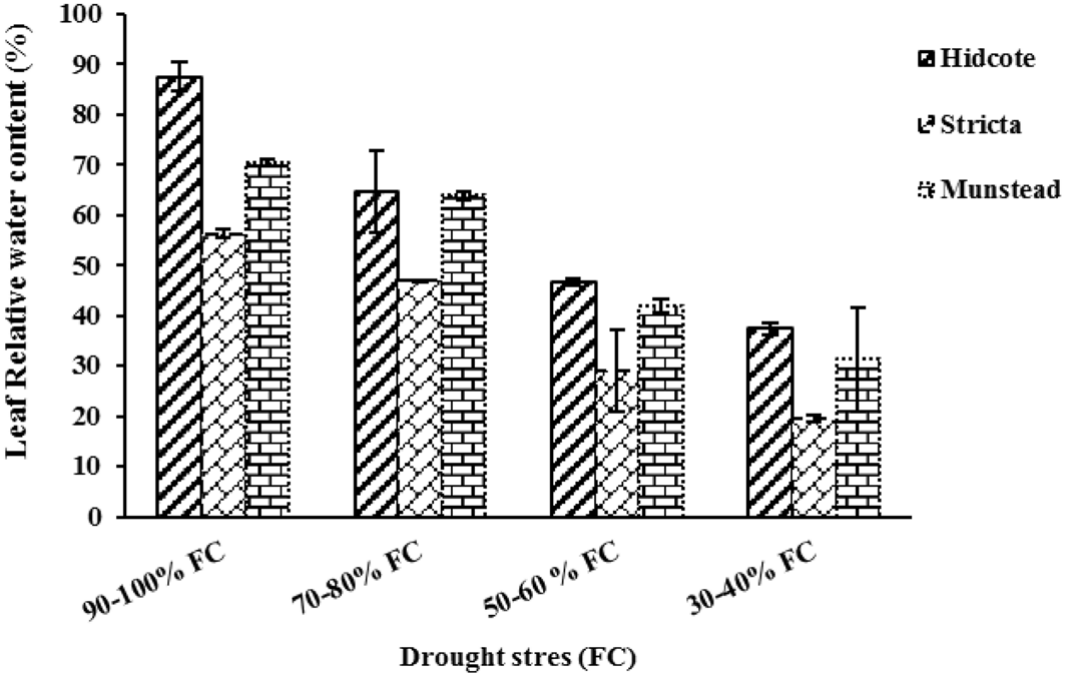
The effect of drought stress on RWC of leaves in different lavender genotypes.

The relative water content (RWC) of leaves was significantly affected by drought stress, genotype and their interaction (Table 2). The highest amount of RWC (87.43%) was observed in H genotype in no drought stress condition. The lowest RWC (19.60%) was observed in S genotype in 30–40% of field capacity (Fig. 4). The results of comparing average data showed that in highest level of drought stress RWC in H, S and M genotypes is 57.25%, 65.19% and 58.88%, respectively; which compared to the control group, it is decreased in all genotypes. This suggests higher resistance of H genotype to maintain RWC of leaves (Fig. 4). In all evaluated genotypes, with increasing drought, RWC was decreased.

RWC is a suitable indicator for water stress in plants. Drought stress by reducing RWC and total water potential of cell, result in reduction in growth of plants. The osmoregulation mechanisms in drought-resistant plants, maintains high RWC in them. Reduction in RWC of leaves as a result of water deficiency stress, is due to reduction in amount of water in tissue, reduction in amount of water in soil, and the negative soil water potential^58^. Alinejad et al. (2020), reported that RWC of leaves in *Datura stramonium* L. plant was decreased due to drought, in a way that the highest amount of RWC (80.22%) was seen in 55% of field capacity, compared to 35% and 15% of field capacity^59^. Also Mohammadi et al. (2018) suggested that RWC of leaves in *Thymu vulgari* L. was decreased to 18.41%, after being exposed to drought^60^.

### Total phenolic and flavonoids contents in leaves

**Figure 5:**
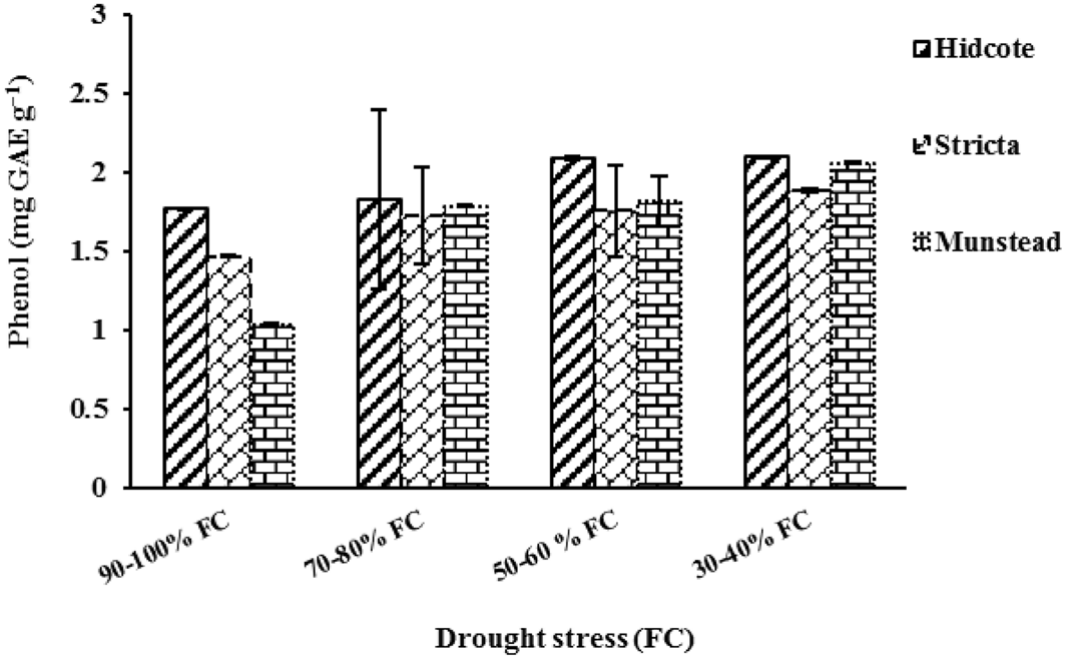
The effect of total phenolic content of leaves in different genotypes of lavender.

Drought, genotype and their interaction had a significant effect on total phenolic content of leaves (Table 2). The results suggest that in different levels of drought, total phenolic content was different in lavender genotypes. In the highest level of drought, total phenolic content in H, S, and M genotypes was respectively increased 18.64%, 28.57% and 98.07% in comparison with the control group. The highest difference in total phenolic content compared to control group was observed in M genotype (Fig. 5).

Total flavonoids content of leaves was significantly (p ≤ 0.01) affected by drought and genotype (Table 2). The results of comparing averages showed that the highest amount of total flavonoids (1.12 mg quercetin per g of fresh weight) was in H genotype, and the lowest amount (0.95 mg quercetin per g of fresh weight) was in M genotype (Table 3). Moreover, our results showed that drought level from I_2_ to I_4_ caused an increase of 12.74%, 14.61% and 15.38% in total flavonoid content of leaves, respectively. Which indicates an increase in flavonoid amount with increasing drought level (Table 3).

**Table 3:** Comparing simple effects of genotype and drought stress on traits of lavender plant.

Treatment	Soluble sugar	Flavonoid	APX^1^
**Genotype**			
*Lavandula angustifolia* cv. Hidecot	40.71^a^	1.12^a^	284.96^a^
*Lavandula stricta*	36.68^b^	0.95^b^	224.21^b^
*Lavandula angustifolia* cv. Muneasted	45.65^c^	0.95^b^	196.08^c^
**Drought stress**			
90–100% FC^2^	25.12^d^	0.918^b^	171.88^c^
70–80% FC	39.29^c^	1.026^a^	227.54^b^
50–60% FC	19.47^b^	1.043^a^	256.39^ab^
30–40% FC	52.45^a^	1.050^a^	284.52^a^

1. APX, Ascorbate peroxidase, 2. Field capacity.

Different letters on top of column indicate significant different at P ≤ 0.05 according to the LSD test.

Total phenolic content is related to stress-resistance, indirectly by helping cell protection, and directly as an antioxidant^61^. Phenolic compounds due to their reductive properties, act as a free radical remover^62^. Our findings are similar to those of a study on growth of *Mentha piperita* in drought stress^54^.

### Total antioxidant activity

**Figure 6:**
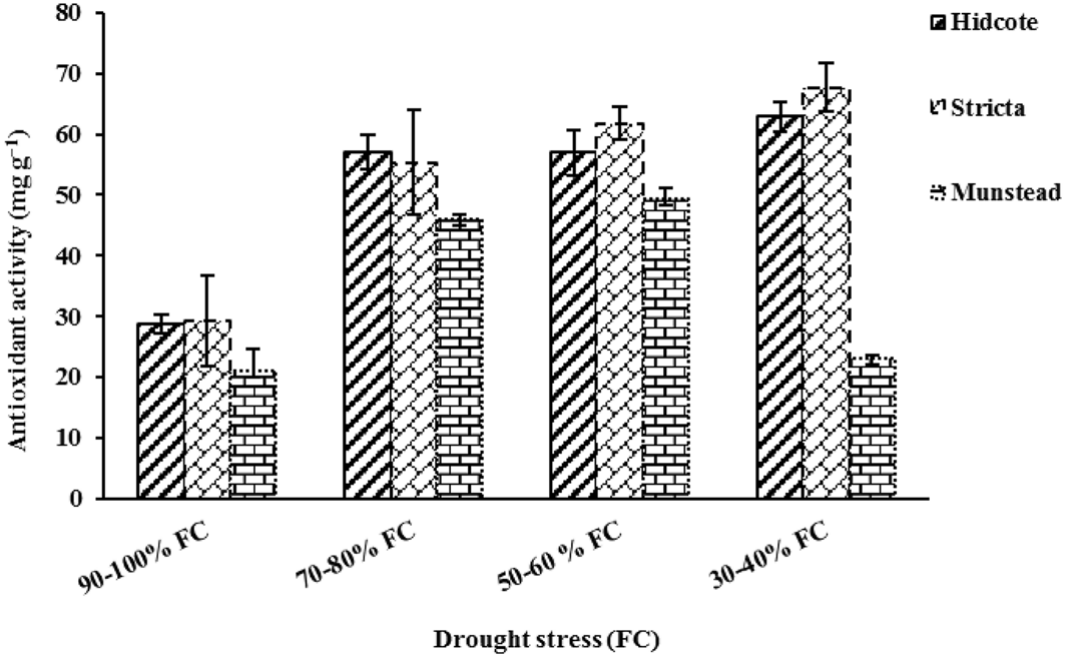
The effect of drought stress on antioxidant activity in different lavender genotypes.

Total antioxidant activity was significantly affected by drought stress and genotype (Table 2). With increasing drought, antioxidant activity in H and S genotypes was increased. The results of comparing average data showed that compared to the control group, in drought levels of I_2_, I_3_ and I_4_, antioxidant activity in H genotype was increased by 98.43%, 98.36% and 118.78%, respectively; and in S genotype this amounts were increased by 89.85%, 111.78%, and 131.90% respectively (Table 5). In M genotype the antioxidant activity has reached its highest amount (49.38 mg/g) in I_3_ level of drought, and then with increasing drought stress the antioxidant activity was decreased, in a way that in highest drought level it had the lowest antioxidant activity (23.18 mg/g). M genotype was used as control (Fig. 6). Our results indicate that in highest drought level, antioxidant activity of S genotype was more than others.

### Antioxidant enzymes

**Figure 7:**
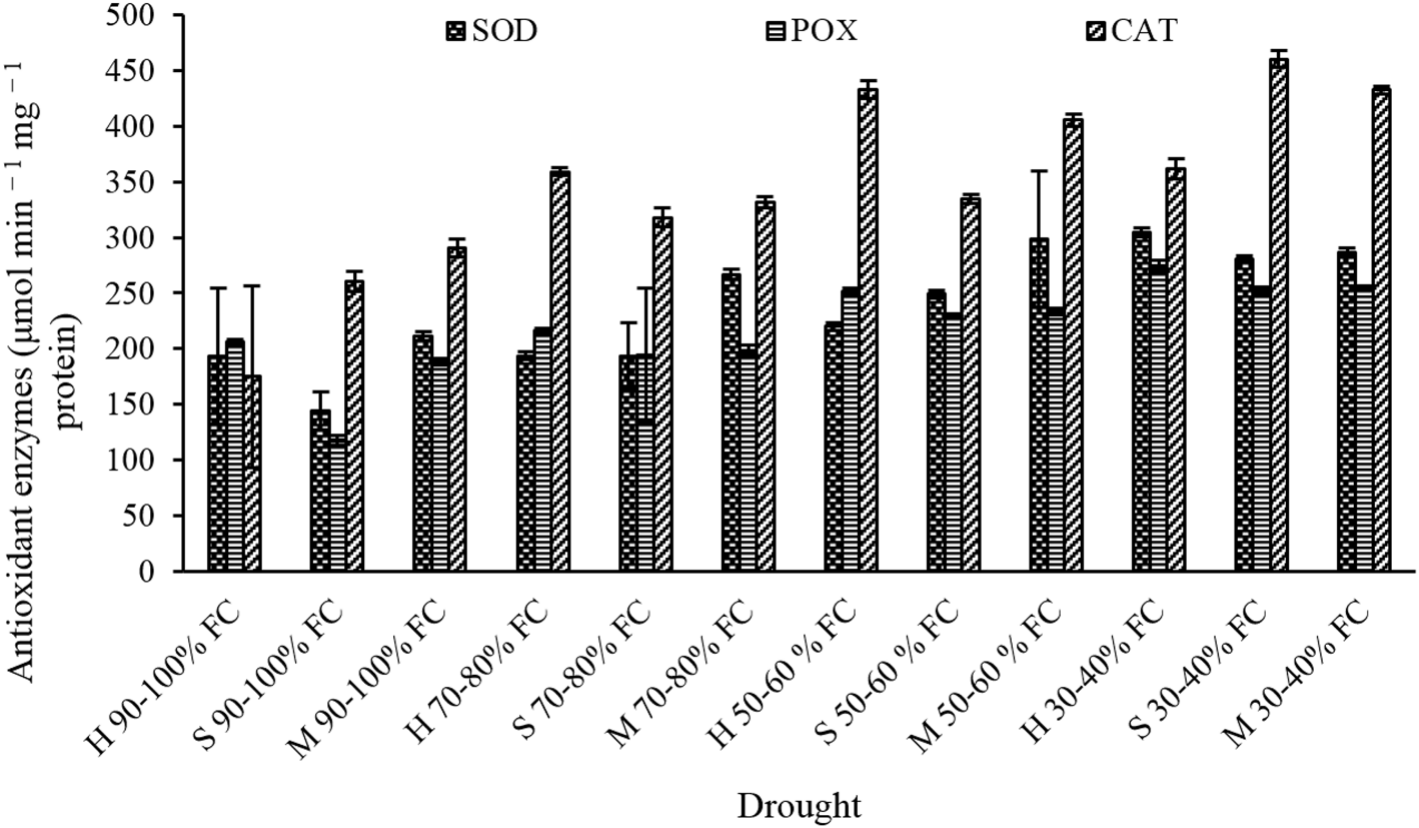
The effect of drought stress on enzymatic activity of SOD, POX and CAT in different lavender genotypes.

Enzymatic activity of antioxidant enzymes in lavender leaves was significantly affected by genotype and drought stress (Table 2). Our results showed that the highest activity of SOD (304.75 μmol min^−1^ mg^−1^ protein) was observed in interaction of H genotype and I_4_ drought level, and the lowest activity of SOD (144.52 μmol min^−1^ mg^−1^ protein) was observed in S genotype with no drought (Fig. 7). Moreover, our observations showed that in I_2_ and I_3_ drought levels, the highest amount of SOD enzymatic activity was related to M genotype (Fig. 7). In the highest drought level, enzymatic activity of SOD was increased in H and S genotypes, and it decreased in M genotype.

Enzymatic activity of peroxidase (POX) enzyme was increased in all three genotypes, with increasing drought. In all drought levels, H genotype had the highest amount of POX activity, compared to other genotypes. There was no significant difference in POX activity in S and H genotypes. The results showed that the highest amount of POX activity (274.48 μmol min^−1^ mg^−1^ protein) was observed in interaction of H genotype and 30–40% field capacity, and the lowest amount (117.66 μmol min^−1^ mg^−1^ protein) was observed in interaction of S genotype and no drought condition (control) (Fig. 7).

Catalase (CAT) enzyme was affected by drought, genotype and their interaction (Table 2). The results of catalase enzyme activity assessment showed that with increasing drought, catalase activity is different in H, M and S genotypes. The most different reaction in production of CAT was related to H genotype, which with increasing drought stress up to I_3_ level, the enzyme activity was increased. But regarding M and S genotypes, with increasing drought level, CAT activity was increased in both genotypes. In this study the highest amount of CAT (460.51 μmol min^−1^ mg^−1^ protein) was observed in interaction of S genotype with 30–40% of field capacity; and the lowest amount (157.06 μmol min^−1^ mg^−1^ protein) was observed in interaction of H genotype with 90–100% of field capacity (Fig. 7).

No significant effect was observed for APX enzyme in interaction of genotype and drought (Table 2). The results of comparing average data, suggest that the highest amount of APX activity (284.96 μmol min^−1^ mg^−1^ protein) was observed in H genotype (Table 3). Also the results showed that I_2_, I_3_ and I_4_ drought level resulted in an increase in APX enzyme activity by 32.38%, 49.16%, and 65.53% respectively. This indicates that APX enzymatic activity increases with increasing drought level (Table 3).

Using physiological and biochemical mechanisms to reduce effects of stress shows that to overcome drought, oxidative stress and to eliminate ROS, plants will increase the amount of antioxidant content^55^. One of major mechanisms to cope with oxidative stress in plants, is activation of antioxidant enzymes^61^. Findings of the present study indicates that different lavender genotypes showed partial resistance against drought. In this research, increased activity of antioxidant enzymes in lavender genotypes under drought condition, was considered as an important drought-resistance factor. Among all antioxidant enzymes, SOD can have a good response against drought stress. In a way that H, S, and M genotypes of lavender in the highest level of drought stress (I_4_), showed an increased amount of SOD, by 57.42%, 35.85% and 60.69% compared to normal conditions (Fig. 7).

In this study, the minimum enzymatic changes were related to the POX enzyme and the highest enzymatic changes were related to the CAT enzyme. Moreover, it was observed that the highest amount of catalase enzymatic activity was in H genotype. In a way that in plants under drought stress CAT activity was increased up to I3 drought level; but, after this level with increasing drought (I4 drought level) CAT enzymatic activity was decreased. CAT and POX are among important plants enzymes which can protect plant cells against free radicals^63^. In this study, in drought period, enzymatic activity of CAT and POX was increased, this means that lavender genotypes, in the face of stress produce antioxidant enzymes to protect themselves. While in H genotype compared to other genotypes, in high drought stress, CAT activity was decreased which this response indicates the different function of this genotype in dealing with ROS. Enzymatic response to drought condition was different in various lavender genotypes. Generally, the negative effect of drought is shown by production of reactive oxygen species (ROS). Increased enzymatic activity of antioxidant enzymes, particularly CAT and POX can reduce the negative effects of drought^64, 65^. In this regard, increased activity of antioxidant enzymes in different genotypes of *Calendula officinalis* plant was reported to^56^.

### Malondialdehyde (MDA) content

**Figure 8:**
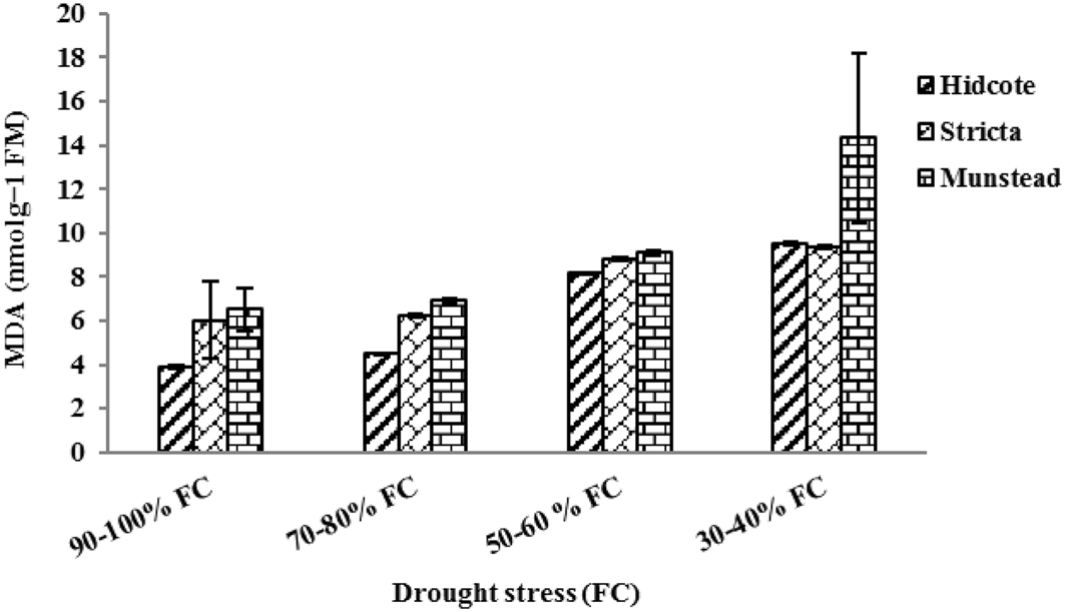
The effect of drought stress on MDA content in different lavender genotypes.

Reaction of different lavender genotypes under drought stress was different in terms of malondialdehyde (MDA) production and accumulation (Table 2). With increasing drought, MDA content was significantly increased in M and H genotypes. The highest amount of MDA in these genotypes was 14.34 and 9.50 nmolg − 1 FM respectively, which was observed in drought level of 30–40% of field capacity. This indicates a significant increase in MDA content with increasing drought (Fig. 8). While the process of production and accumulation of MDA in S genotype was different at various drought levels. For S genotype, in first level of drought (I_2_), MDA content was increased which showed the vulnerability of the cell membrane at this drought level. But with increasing drought, gradually, the S genotype plants adapted to the dry environment, which in this level cell membrane damage was not obvious. Then, increasing in drought stress resulted in increased MDA content. Generally, in I_2_ and I3 drought levels, lavender genotypes underwent varying degrees of damage, which in M and H genotypes followed by increasing enzymatic activity, and in S genotype it resulted in decreased enzymatic activity. But in the highest level of drought (I_4_), the cell membrane was seriously damaged and in all three genotypes and MDA content was significantly increased (Fig. 8).

Membrane lipid peroxidation due to the accumulation of active oxygen species leads to cell damage and death. In plants this lipid peroxidation happens under drought stress^66^. MDA is the final product of membrane peroxidation and membrane processes. Simultaneously with peroxidation, the MDA content increases significantly^67^. So the MDA content can be considered as an indicator of drought-resistance in plants. Among lavender genotypes, in the highest level of drought, MDA content in M genotype was significantly increased compared to others genotypes; whish suggests that M genotype is more vulnerable in comparison with the two other genotypes. An increase in MDA content under drought stress, was reported in Thymus species as well^66^.

### Quantity and quality of essential oil

**Figure 9:**
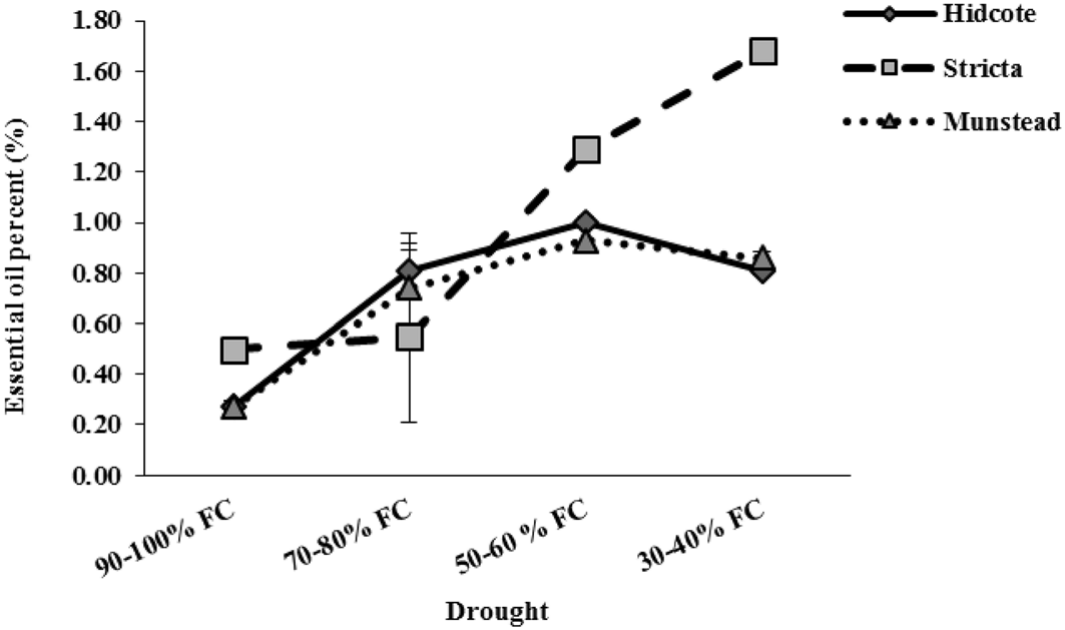
The effect of drought stress on essential oil percent in different lavender genotypes.

Mutual interaction between drought stress and genotype had a significant effect on percentage and yield of essential oil in lavender plants (Table 2). Our findings suggested a different essential oil percentage for each genotype in various levels of drought stress. With increasing drought to I_3_ level, the essential oil percentage was increased in M and H genotypes, but after that with increasing drought to a higher level (I_4_), essential oil percentage in these genotypes was decreased. While in S genotype, increasing essential oil percentage totally had an upward trend (Fig. 9).

Evaluation of essential oil percentage in different levels of drought, showed that in I_2_ drought level, the highest amount of essential oil (0.81%) was observed in H genotype; and in I_3_ and I_4_ drought levels, the highest amounts of essential oil were 1.29% and 1.68% respectively, which were observed in S genotype. Moreover, our results suggest that the highest difference in essential oil percentage in the studied genotypes compared to the control, was related to S genotype (Fig. 9). Totally, the highest percentage of essential oil was observed in S genotype in I_4_ drought level. This shows the high capacity of this genotype to produce essential oil under drought stress.

**Figure 10:**
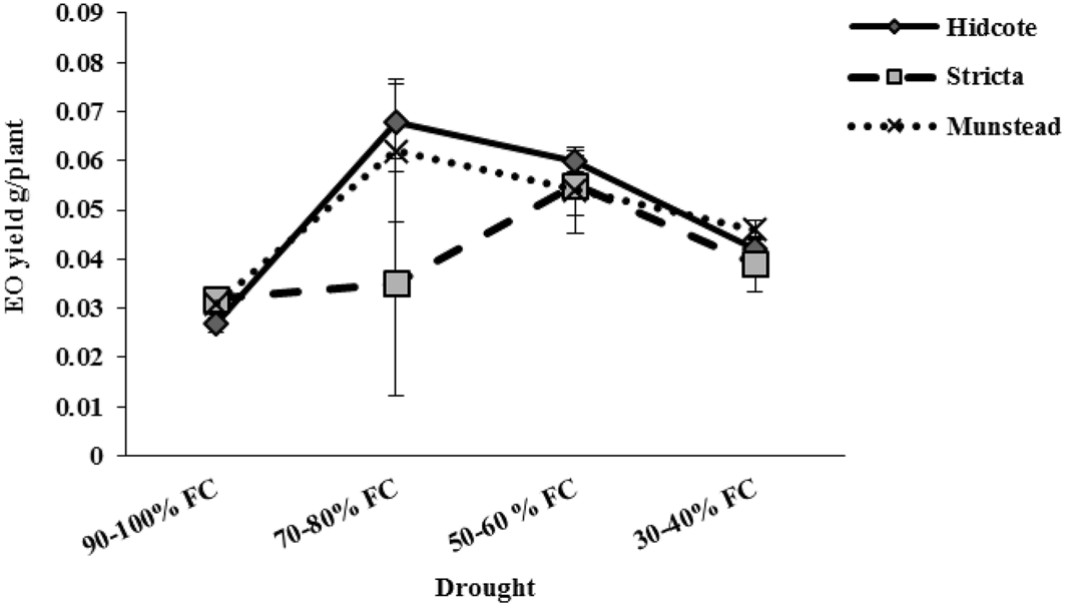
The effect of drought stress on essential oil yield in different lavender genotypes.

Essential oil yield was significantly affected by genotype and drought. The results showed that the essential oil yield in S genotype was different from the others. So that the highest yield of essential oil (0.055 g per plant) was observed in this genotype in I_3_ drought level. While in H and M genotypes the highest amounts were 0.068 g and 0.065 g respectively, which were gained in I_2_ drought level (Fig. 10). Results of comparing average data showed that the highest yield of essential oil at I_2_ and I_3_ levels was obtained with 151/85% and 122.22% difference compared to the control, respectively, and they gained from H genotype. This indicates the high potential of H genotype to maintain biomass and produce essential oil in drought stress. Also our results suggest that in the highest drought level (I_4_), the highest essential oil yield (0.046 g per plant) was observed in M genotype (Fig. 10).

### Principal component analysis (PCA)

**Figure 11:**
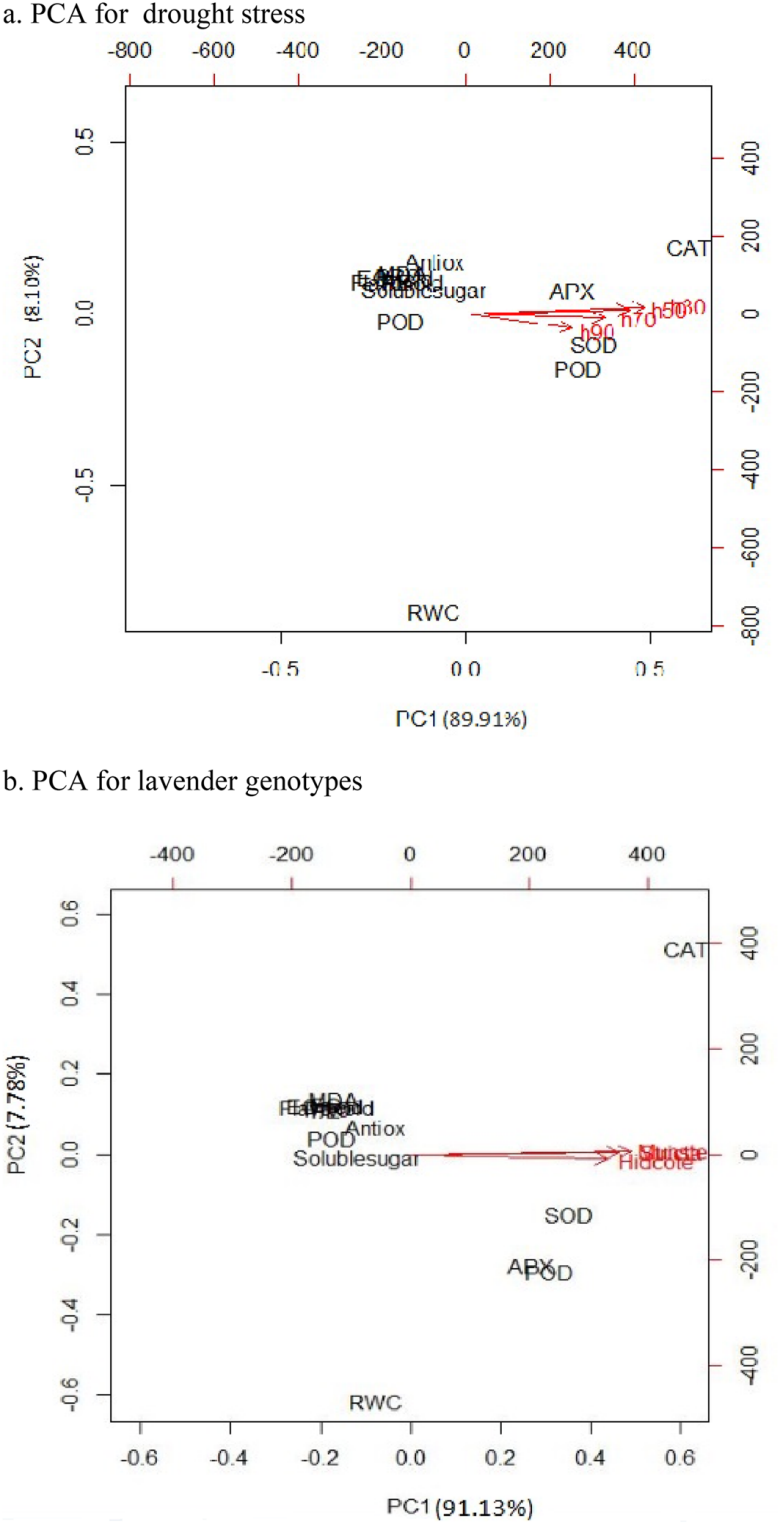
Principal component analysis (PCA) for genotypes (**a**) and physiological traits (**b**) based on water status calculated for physiological traits. (R 4.0.4 packages, https://rstudio.com/products/rstudio/).

PCA analysis was performed to identify susceptibility of genotypes in irrigation regimes. According to physiological traits in the PCA analysis (Fig. 11a, b), the first factor (PC1) explains about 90% of the total variance of variables, and the second factor (PC2) about 8%.

The results of PCA analysis of different irrigation regimes showed that in the first component, which shows 89.91% of changes, the best traits are antioxidant enzymes CAT, SOD, APX, while in the second component, with 8.10% changes, only the trait Catalase is the best trait. Also, in total, the first and second components, which show 98.01% of the changes, show CAT as the most effective trait (Fig. 11a).

The results of PCA analysis in lavender genotypes showed that the first and second main components could explain 98.91% of the existing changes. So that the first main component with 91.13% and the second component with 7.78% had a share in the total variation. Therefore, using these two components and ignoring other components will only cause the loss of a small part of about 1.09% of the data changes (Fig. 11b). These two principal components include peroxidase, ascorbate peroxidase, and superoxide. Physiological responses of *Lavandula* genotypes (*L. angustifolia* cv. Hidcote, *L. angustifolia* cv. Munstead, and *L. stricta*) submitted to drought stress were evaluated through principal component analysis (PCA), and the results are illustrated in Fig. 11a. *Lavandula stricta* presents higher levels of CAT activity than *L. angustifolia* cv. Hidcote and *L. angustifolia* cv. Munstead. In addition, APX and CAT increase in stress-treated in 30–40% FC. This result shows that *L. stricta* exhibits the most affected physiological changes while trying to adjust to changes in the water status of the environment, under the imposed conditions and shows the highest resistance.

The results of analysis of essentials oils from H, S and M genotypes is shown is Tables 4, 5 and 6. The trend of changes in essential oils composition is described in all three genotypes. By studying the mass spectra and the Kovats retention index, 23 compounds were identified in the H genotype’s essential oil (Table 4). The yield of H genotype essential oil from I_1_ to I_4_ drought levels was 99.89%, 82.78%, 81.09% and 82.85%, respectively. The main components of H genotype essential oil in I_1_ to I_4_ drought levels, include 1.8-Cineol compounds (5.94%, 7.73%, 4.24% and 3.50%), Linalool (23.20%, 16.30%, 11.90% and 10.57%), Camphor (3.41%, 4.65%, 2.32% and 2.87%), Borneol (4.89%, 3.34%, 3.65% and 3.01%), Bornyl formate (27.32%, 16.04%, 19.45% and 20.03%), Lavandulyl acetate (1.40%, 4.21%, 6 and 8.35%), Caryophyllene oxide (10.92%, 11.77%, 12.16% and 19.91%), α-Muurolene (4.38%, 3.20%, 1.20% and 0%) (Table 4). The results of grouping the essential oil compounds showed that the amount of hydrocarbon monoterpenes from I_1_ to I_4_ drought level were 12.88%, 8.86%, 8.53% and 6.06%, respectively. The amount of oxygen monoterpenes was 64.76%, 50.70%, 43.32% and 42.45%; and hydrocarbon sesquiterpene compounds were 13.12%, 11.45%, 13.03% and 13.96%. The amount of oxygen sesquiterpene compounds were 10.92%, 11.77%, 16.21%, and 19.91%; which shows that increasing drought level, result in decreasing monoterpene compounds, and increasing sesquiterpene compounds.

**Table 4:** Chemical composition of essential oils extracted from *Lavandula angustifolia* cv. Hidcote plants under different irrigation regime.

No	Compound	RI^1^	Area %			
			I_1_	I_2_	I_3_	I_4_
1	α-thujene	926	0.26	–	–	0.25
2	α-Pinene	933	2.01	–	2.80	0.95
3	Camphene	948	1.21	1.78	2.10	2.48
4	Sabinene	971	0.34	0.28	0.26	–
5	β-Pinene	977	0.74	0.45	0.30	–
6	δ-3-Carene	1011	3.02	2.92	1.80	0.89
7	m-Cymene	1022	–	–	–	–
8	p-Cymene	1024	2.44	1.78	1.27	0.93
9	O-Cymene	1025	2.06	1.65	-	0.56
10	1,8-Cineol	1032	5.94	7.73	4.24	3.5
11	Linalool	1102	23.20	16.30	11.90	10.57
12	Camphor	1145	3.41	4.65	2.32	2.87
13	Borneol	1170	4.89	3.34	3.65	3.01
14	Bornyl formate	1227	27.32	16.04	19.45	20.03
15	Linalyl acetate	1259	–	2.64	0.76	2.47
16	Lavandulyl acetate	1290	1.40	4.21	6.00	8.35
17	Myrtenyl acetate	1341	0.83	1.16	1.26	1.36
18	α-Santalene	1419	2.50	2.30	1.56	1.67
19	Caryophyllene	1430	–	–	–	0.56
20	trans-β-Bergamotene	1436	1.68	0.76	1.12	0.46
21	γ-Cadinene	1514	1.34	–	1.88	1.56
22	Caryophyllene oxide	1584	10.92	11.77	16.12	19.91
23	α-Muurolene	1643	4.38	3.02	1.21	–
	Total		99.89	82.78	81.09	82.58
	Monoterpene Hydrocarbons		12.08	8.86	8.53	6.06
	Oxygenated Monoterpene		64.76	50.70	43.32	42.45
	Sesquiterpene Hydrocarbons		12.13	11.45	13.03	13.96
	Oxygenated sesquiterpenes		10.92	11.77	16.21	19.91

1. Retention Index, I_1_, 100–90% of field capacity (control)-Irrigation Regime 1; I_2_, 80–70% of field capacity-Irrigation Regime 2; I_3_, 60–50% of field capacity-Irrigation Regime 3; I_4_, 30–40% of field capacity-Irrigation Regime 4.

**Table 5:** Chemical composition of essential oils extracted from *Lavandula stricta* plants under different irrigation regime.

No	Compound	RI	Area %			
			I_1_	I_2_	I_3_	I_4_
1	α-Thujene	926	–	–	0.34	0.90
2	α-Pinene	940	2.84	3.20	2.31	1.77
3	Limonene	1036	2.5	2.24	1.92	1.67
4	Linalool	1107	32.6	28.45	20.12	19.21
5	Amyl isovalerate	1120	3.17	2.19	0.86	–
6	Citronellol*	1181	3.8	2.78	1.10	–
7	decanal	1229	10.26	15.21	18.56	19.72
8	1-Decanol	1288	8.01	10.31	17.88	21.34
9	β-Ionone	1490	2.65	2.09	1.05	0.18
10	Sesquiphellandrene < beta- >	1525	2.24	2.22	2.76	2.81
11	Kessane	1529	2.44	4.43	9.99	11.5
12	Caryophyllene oxide	1585	1.33	2.81	3.74	3.19
13	Hexadecane	1596	1.26	5.77	6.10	9.11
14	α-Muurolene	1645	2.19	2.28	2.99	3.32
15	Aromadendrene oxide	1665	2.12	2	1.07	–
16	2-methyl-1-hexadecanol	1681	11.1	9.32	8.15	2.37
17	Hexahydrofarnesyl acetone	1848	6.8	6.34	3.78	1.26
18	Isobutyl phthalate	1876	4.1	3.19	2.71	–
	Total		99.41	98.48	99.53	99.93
	Monoterpene Hydrocarbons		5.34	5.44	4.57	4.34
	Oxygenated Monoterpene		60.49	61.03	59.57	60.45
	Sesquiterpene Hydrocarbons		5.69	10.27	11.85	15.24
	Oxygenated sesquiterpenes		27.89	28.09	29.44	18.32

**Table 6:** Chemical composition of essential oils extracted from *Lavandula angustifolia* cv. Munstead plants different irrigation regime.

No	Compound	RI	Area %			
			I_1_	I_2_	I_3_	I_4_
1	Tricyclene	922	0.23	0.17	–	–
2	α-Pinene	933	1.54	1.53	1.12	0.92
3	Camphene	950	7	6.88	5.32	3.21
4	Thuja-2,4(10)-diene	954	0.16	0.10	–	–
5	β-Pinene	978	2.79	2.80	2.11	1.67
6	δ-3-Carene	1011	0.56	0.45	0.34	0.19
7	p-Cymene	1022	1.41	1.34	1.46	1.13
8	O-Cymene	1026	3.03	3.18	2.78	2.33
9	Limonene	1029	1.2	1	0.78	0.51
10	1,8-Cineol	1031	0.51	0.50	0.42	0.26
11	Camphor	1150	16.82	16.32	17.11	18.30
12	Pinocarvone	1164	0.51	0.46	0.31	0.16
13	Borneol1	1179	44.96	42.80	37.54	30.99
14	4-Terpineol	1184	0.16	0.10	–	–
15	p-Cymen-8-ol	1191	1.2	1.11	0.98	0.47
16	Myrtenal	1201	0.29	0.21	0.14	–
17	Myrtenol	1203	0.2	0.11	–	–
18	Eucarvone	1214	0.27	0.23	0.16	0.11
19	Bornyl formate	1229	0.26	0.22	0.16	–
20	Bornyl Acetate	1286	0.3	0.21	0.14	0.13
21	Myrtenyl acetate	1340	0.11	0.10	–	–
22	α-Santalene	1420	0.26	0.89	1.43	2.54
23	γ-Cadinene	1515	0.17	0.89	1.34	2.10
24	δ-Cadinene	1523	0.27	0.34	0.98	1.32
25	Caryophyllene oxide	1590	14.68	15.21	15.90	17.21
26	α-Muurolene	1645	0.88	1.11	1.67	2.13
27	Ledene oxide-(II)	1677	0.23	0.44	0.89	1.16
	Total		99.90	98.38	93.08	87.04
	Monoterpene Hydrocarbons		17.82	17.45	13.91	9.96
	Oxygenated Monoterpene		65.59	62.05	56.96	50.42
	Sesquiterpene Hydrocarbons		1.58	3.23	5.42	8.09
	Oxygenated sesquiterpenes		14.91	15.65	16.79	18.37

**Figure 12:**
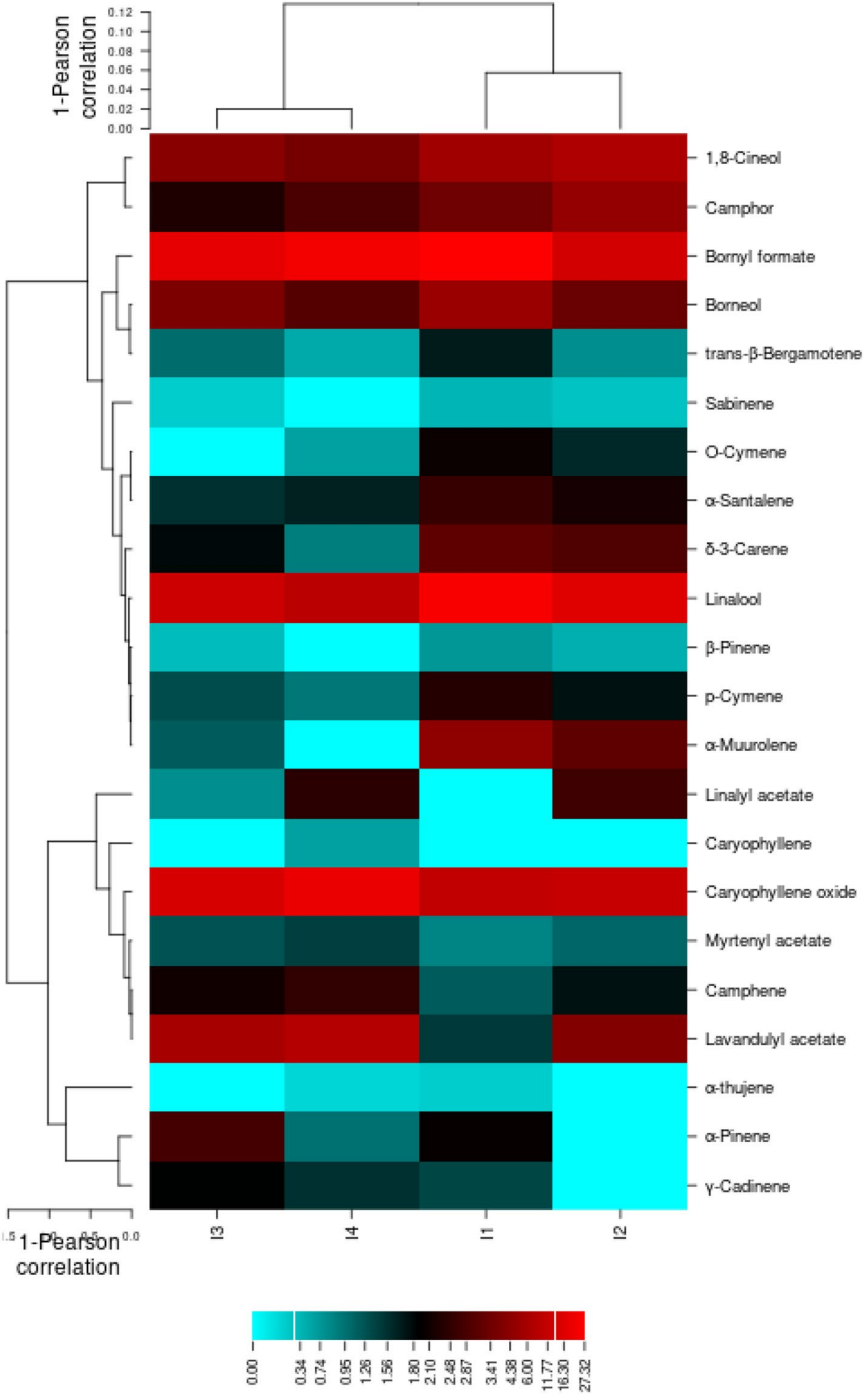
Heatmap for the essential oil profile in aerial parts of *Lavandula angustifolia* cv. Hidcote corresponding to irrigation regimes (CIMminer, https://discover.nci.nih.gov/cimminer/oneMatrix.do).

Heat map for the essential oil profile in *Lavandula angustifolia* cv. Hidcote corresponding to the different irrigation regime The similar discrimination was also supported by the heatmap constructed for essential compounds. Accordingly, 22 rows and 4 columns were achieved. α- pinene, β-Pinene, δ-3-Carene, type of Cymene, 1,8-Cineol, Camphor and Linalool from the main compounds, peaked at control. Moreover, lavandulyl acetate, Myrtenyl acetate, caryophyllene oxide, camphene and γ-Cadinene revealed highest percentage at 30–40% FC, Some compounds, such as Camphor and Linalyl acetate, are at the levels of the intermediate irrigation regime (Fig. 12). It is remarkable that as the water limit increases, the amount of monoterpene compounds decreases and the amount of sesquiterpene compounds increases.

With evaluation of the essential oil from S genotype, 18 compounds were identified (Table 5). The amount of essential oil in I_1_ to I_4_ drought levels was 99.41%, 98.48%, 99.53% and 99.93% respectively (Table 5). Among identified compounds in S genotype the followings were accounted for the highest amount of components in the essential oil in I_1_ to I_4_ levels respectively; Linalool (32.60%, 28.45%, 20.12% and 19.12%), decanal (10.26%, 15.21%, 18.56% and 19.27%), 1-Decanol (8.01%, 10.31%, 17.88% and 21.34%), Kessane (2.44%, 4.43%, 9.99% and 11.50%), Hexadecane (1.26%, 5.77%, 6.10% and 11.9%), 2-methyl-1-hexadecanol (11.1%, 9.32%, 8.15% and 2.37%) and Hexahydrofarnesyl acetone (6.8%, 6.34%, 3.78% and 1.26%) (Table 5). The most obvious point was the high percentage of Linalool, decanal and 1-Decanol in the S genotype. With increasing drought, Linalool compounds were decreased and decanal and 1-Decanol compounds were increased. The grouping of essential oil components also showed that among the 18 compounds identified, the following were the highest in I_1_ to I_4_ drought levels, respectively; 3 hydrocarbon monoterpenes with total of (5.34%, 5.44%, 4.57% and 4.34%), 6 oxygen monoterpenes with total of (60.49%, 61.03%, 59.57% and 60.45%), 3 hydrogen sesquiterpenes with total of (5.69%, 10.27%, 11.85% and 15.24%) and 6 oxygen sesquiterpenes with total of (27.89%, 28.09%, 29.44% and 18.32%). With increasing drought, the amounts of hydrocarbon monoterpenes and oxygen sesquiterpenes were decreased; while the amount of hydrocarbon sesquiterpenes was increased. Also the highest amount of oxygen monoterpenes, by 61.03%, was seen in I_2_ drought level.

**Figure 13:**
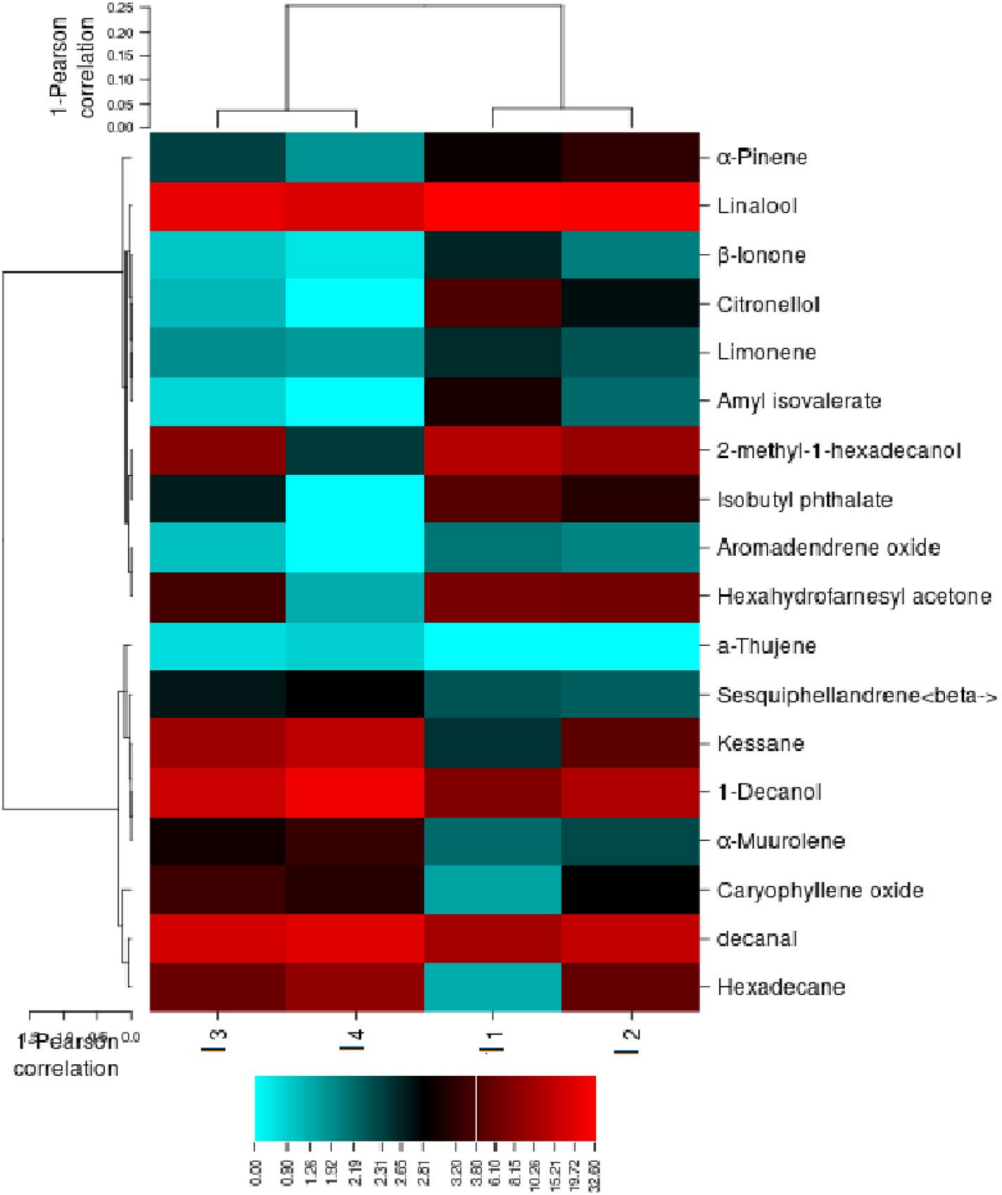
Heatmap for the essential oil profile in aerial parts of *Lavandula stricta* corresponding to irrigation regimes (CIMminer, https://discover.nci.nih.gov/cimminer/oneMatrix.do).

Heat map for the essential oil profile in *Lavandula stricta* corresponding to the different irrigation regime The parallel discrimination was also supported by the heatmap constructed for essential compounds. Accordingly, 18 rows and 4 columns were achieved. α- pinene, Amyl isovalerate, Citronellol, β-Ionone and Linalool from the main compounds, peaked at control. Moreover, α-Thujene, decanal, 1-Decanol, Sesquiphellandrene, Kessane and Hexadecane revealed highest percentage at 30–40% FC (Fig. 13). These results confirm the results obtained from the *Lavandula angustifolia* cv. Hidcote so that as the water limit increases, the amount of monoterpene compounds decreases and the amount of Sesquiterpene compounds increases.

Essential oil yield in M genotype from I_1_ to I_4_ drought levels was obtained 99.90%, 98.38%, 93.08% and 87.04% (Table 6). As it is shown in Table 6, analysis of the essential oil from M genotype included 27 compounds which its major part was consisted of Camphor (16.82%, 16.32%, 17.11% and 18.30%), Borneol (44.96%, 42.80%, 37.54% and 30.99%) and Caryophyllene oxide (14.68%, 15.21%, 15.90% and 17.21%) from I_1_ to I_4_ drought levels, respectively. comparison of essential oil components (Table 6) showed that from 27 identified compounds in M genotype, the followings were the most prevalent from I_1_ to I_4_ levels respectively, including hydrocarbon monoterpene with total of (17.82%, 17.45%, 13.91% and 9.96%), 12 total oxygen monoterpene compounds with total of (65.95%, 62.05%, 56.96% and 50.42%), 4 hydrocarbon sesquiterpenes with total of (1.58%, 23.23%, 5.42% and 8.09%) and 2 oxygen sesquiterpenes with total of (14.91%, 15.65%, 16.79% and 18.37%). The highest drought level resulted in 31.76% and 17.23% increase in Camphor and Caryophyllene oxide. It also caused 31.07% decrease in Borneol compared to the control (Table 6). Totally, with increasing drought level, monoterpene compounds were decreased and sesquiterpene compounds were increased in lavender genotypes.

The major components of essential oil were different in various lavender genotypes in the highest level of drought (I_4_). In this study in H genotype, the compounds Linalool, Bornyl formate and Caryophyllene oxide; in S genotype the compounds Linalool, decanal, 1-Decanol, Kessane and Hexadecane; and in M genotype the compounds Camphor, Borneol and Caryophyllene oxide, were the most prevalent components of essential oil. In this study, Borneol compound was not observed in S genotype. regarding the fact that essential oil extraction was performed on flowering branches in all three genotypes, and they were studied under similar drought conditions; and also comparing the results of this study with finding of other studies shows that the difference in types and percentage of essential oil’s components can be due to the effect of genetic differences; and to some extent, environmental factors on essential oil in different genotypes.

A total comparison of essential oil analysis results for different lavender genotypes under drought stress showed that oxygen monoterpenes are the most prevalent components of the essential oil, which will decrease with increasing drought level. Sarker et al. (2012) reported that the essential oil of lavender (*Lavandula angustifolia*) contains high amounts of linalool and linalool acetate, along with scares amount of other monoterpenes^68^. A study by Hassan et al. (2014) showed that the compounds carvacrol, phenol-2-amino-4, 6-bis, trans-2-caren-4-ol, and n-hexadecanoic acid are the main constituents of *Lavandula stricta* plants which were collected from the Shaza Mountains in southern Saudi Arabia^69^. Total results from essential oil analysis in this study showed that Linalool was the main ingredient of essential oils in H and S genotypes. This compound is an oxygen monoterpene with a density of 0.85 and a pleasant smell, and is the main component of the essential oil from lavender plant. While in M genotype, Borneol was the main component of the essential oil, which is a circular monoterpene compound with density of Mohammadnejad ganji et al. (2017) suggested that the difference in natural quality of the essential oil from lavender plants is related to intrinsic factors (genetic or heredity capabilities and maturity), and external factors including sunlight, water, heat, pressure, latitude, and soil which affect plant growth and essential oil production^70^.

**Figure 14:**
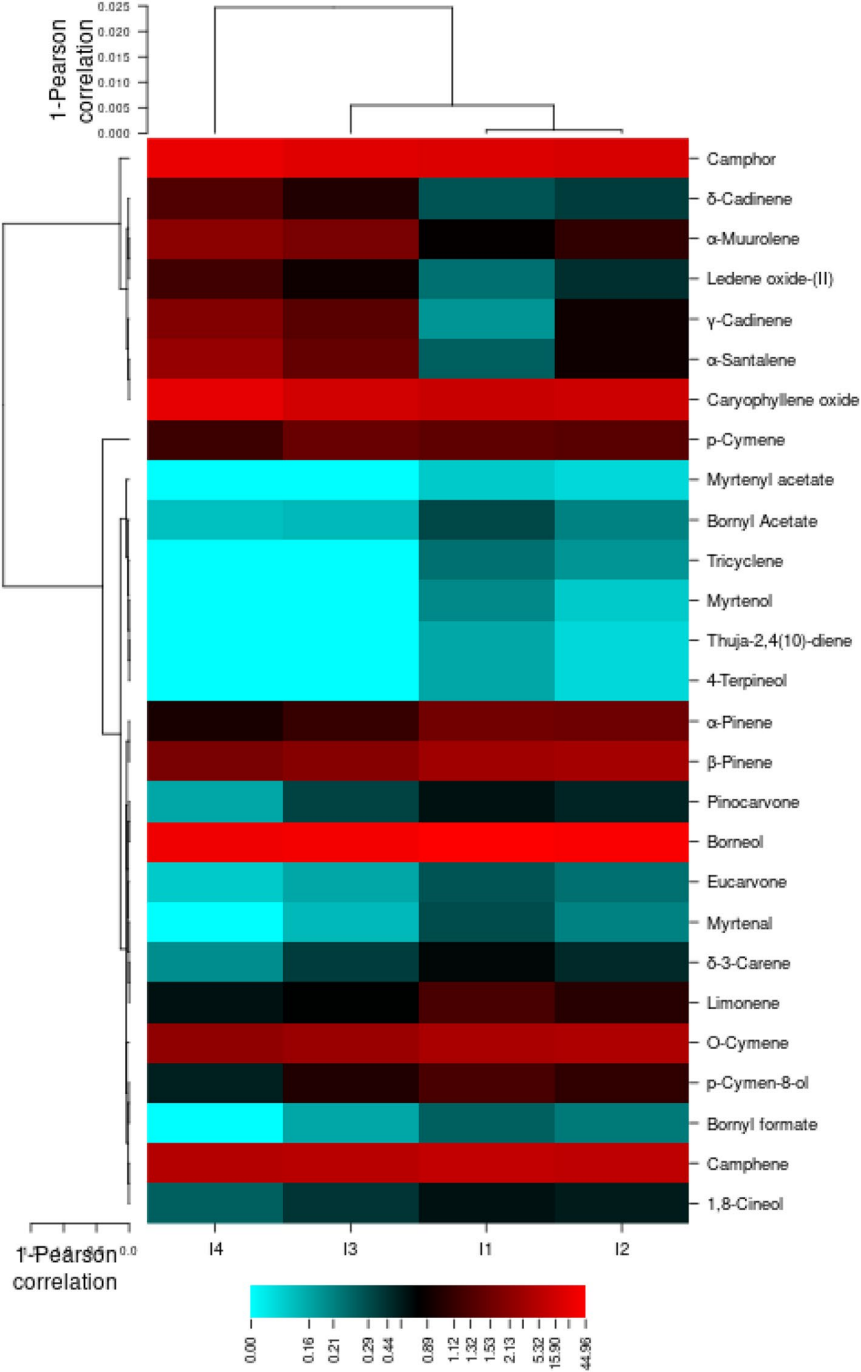
Heatmap for the essential oil profile in aerial parts of *Lavandula angustifolia* cv. Munstead corresponding to irrigation regimes (CIMminer, https://discover.nci.nih.gov/cimminer/oneMatrix.do).

Heat map for the essential oil profile in *Lavandula angustifolia* cv. Munstead corresponding to the different irrigation regime The parallel discrimination was also supported by the heatmap constructed for essential compounds. Accordingly, 18 rows and 4 columns were achieved. α- pinene, Tricycle, Camphene, Thuja-2,4(10)-diene, δ-3-Carene, ρ-Cymene, Borneol and limonene from the main compounds, peaked at control. Moreover, Camphor, α-Santalene, γ-Cadinene, δ-Cadinene, Caryophyllene oxide, α-Muurolene and Ledene oxide-(II) revealed highest percentage at 30–40% FC (Fig. 14). The results showed that the composition of the compounds was similar to the previous two genotypes and the water limit increases, the amount of monoterpene compounds decreases and the amount of Sesquiterpene compounds increases.

Essential oils are generally in the group of terpenoids and The structure of terpenoids consists of two main precursors, isopentenyl pyrophosphate (IPP) and its isomer, dimethylallyl pyrophosphate (DMAPP). These compounds are synthesized via the cytosolic pathway of mevalonic acid (MVA) or plasticity of methylerythritol phosphate (MEP)^71^. The MVA pathway is primarily responsible for the synthesis of Sesquiterpenoids and triterpenoids, while the MEP pathway is used for the biosynthesis of monoterpenoids, diterpenoids and tetraterpenoids^72^. Monoterpenes and Sesquiterpenes are the main constituents of essential oils that play a role in aroma, flavor, photosynthetic pigments and antioxidant activities^73^.

In drought conditions, the amount of these isoprenes does not decrease in relation to the mediators of the MEP pathway and in contrast sometimes increases. Therefore, sesquiterpene compounds increase in drought conditions because most of these compounds are synthesized through the MVA pathway^74^. Another reason for the decrease in MEP path flux is the location of this path, which has a significant impact in drought conditions. In this case, plastids are not able to provide the required IPP of this path, so most monoterpene compounds are reduced^75^.

Also, since the quality of the essential oil is due to the presence of linalool and linalyl acetate^76^. According to the results obtained from heatmaps related to essential oils, three genotypes are identified, the highest amount of linalool amount in S genotype was remained under mind- (I2) till severe-drought (I4) condition. This indicates more compatibility with maintaining the desired quality of drought conditions in this plant than the other two commercial genotypes. And then the H genotype is in the second stage due to the presence of important compounds.

Comparing the grouping created in the heat maps related to the essential oil of 3 genotypes, it is clear that the two genotypes S and H were divided into two groups I1, I2 and I3, I4 in the genotype. But in the genotype M, the results were divided into I4 and I3 groups I2 were divided into genotypes. This can be due to differences in the resistance mechanism of plants in different genotypes, so in genotypes S and H of the plant through increasing sesquiterpene compounds showed resistance to drought stress, while in genotype M increased resistance to drought levels through higher monoterpene compounds. Another conclusion that can be drawn from these heat maps is that in genotypes S and H, the rate of drought resistance in the first and second levels of drought with the third and fourth levels has shown more changes in the type of essential oil compounds, while in the third genotype (M) these changes in the last level drought has been most evident.

At a glance, it seems Genotype S has a different mechanism in reducing the negative effects of drought compared to genotypes M and H, So that, among the enzymatic and non-enzymatic mechanisms, it tends to use the enzymatic pathway more. In association with the production of "proline", drought stress index osmolyte, genotype S has a different trend from genotypes H and M and this osmolyte in this plant has a lower production flux compared to other genotypes. Also, due to the fact that the production of soluble sugars in this plant has been moderate compared to other genotypes, it is expected this genotype replace proline with another osmolyte or uses an enzymatic mechanism to deal with drought, as the results of antioxidant enzyme "catalase" related to genotype S had the highest value with a significant difference under drought stress, while, in the H and M genotypes, the SOD enzyme was responsive to drought.

**Figure 15:**
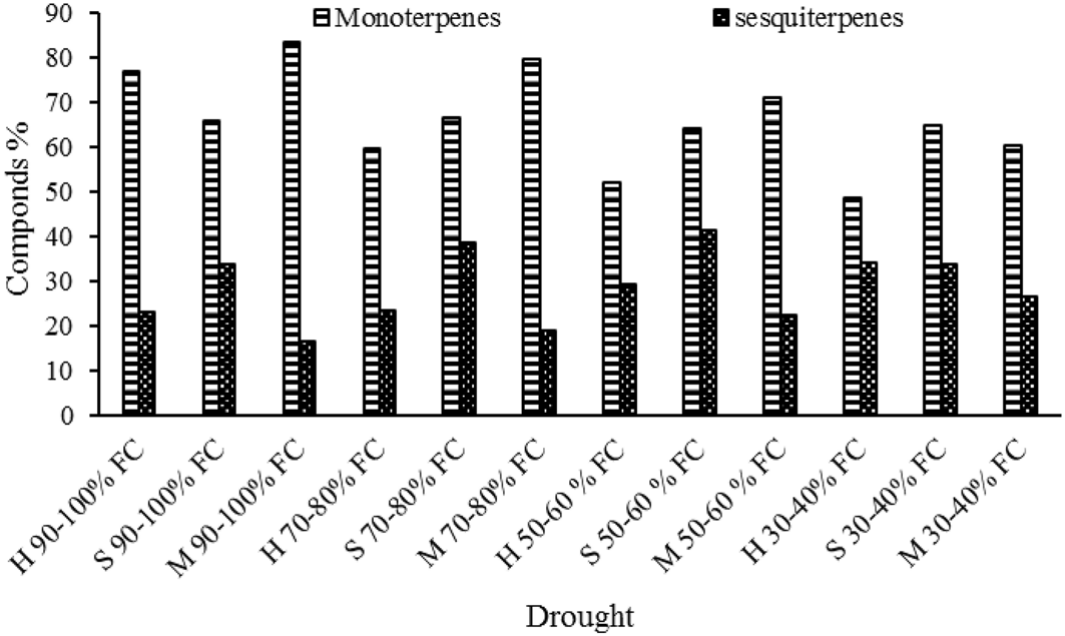
The amount of monoterpenes and sesquiterpene compounds in different genotypes under irrigation regimes.

On the other hand, the high resistance of genotype S can be attributed to the greater activation of the pathway of essential oil compounds. Because by examining the constituents of the essential oil (monoterpene and sesquiterpene), it can be concluded that genotype H and then M at high drought levels still retain the ability to produce monoterpene compounds, while in genotype S with increasing drought, the amount of semi-heavy compounds (sesquiterpene) has increased significantly (Fig. 15), this can confirm the existence of a different resistance mechanism in the S genotype. Because some structural compounds of the membrane, such as sterols, are made from the mevalonic acid (MVA) pathway of acetyl coenzyme A origin. For this reason it seems that S genotype by setting up terpenoid pathways involved in the production of steroids another solution to drought is by preserving its plasma membrane. Steroids are derivatives of triterpenes that, along with phospholipids, are major components of plasma membranes^70^. Also, the study of MDA content as the final product of membrane lipid peroxidation in genotypes at the fourth level of drought (the most severe drought) showed the M genotype is most sensitive to drought. In this way, the two genotypes S and H have almost equal MDA content, so that it can be said that with a small difference from genotype S, genotype H has less composition.

Continuous production of isoprene under drought conditions shows that despite the reduction in the synthesis of osmolyte and relative increasing of MDA (with very little difference from genotype H) that occurs under these conditions, the function of this pathway is essential for the S genotype. Isoprene has long been used to protect plants from drought, high temperatures and oxidative stress are recommended^77^. Of course, it was showed which is possible with increasing drought, sufficient isoprene is not produced to counteract and launch defense pathways and instead used as a general signal to increase drought tolerance^78,79^.

Reasons such as further activation of terpenoid skeletal pathways towards the production of semi-heavy (sesquiterpene) compounds, production of steroids via the MVA pathway could be a reason for lower susceptibility of S genotype and high resistance of this genotype through these mechanisms compared to other genotypes. In contrast, on the one hand, H genotype using proline production, soluble sugar levels and decreased MDA in response to stress caused by drought and on the other hand, the ability to produce substances important monoterpenes, such as Linalool and Linalyl acetate, with the aim of using medicine and aromatherapy^76^, It (H genotype) can be considered as a cultivar with high commercial value and significant resistance to M genotype.

## Conclusion

Considering the results of the present study, the effect of drought in changing quantity and components of the essential oil is confirmed. So that *L. stricta* genotype had the highest percentage of essential oil. Also *L. stricta* contained the highest percentage of Linalool, compared to other genotypes. Since climate change and subsequent drought are among the most important threats to biodiversity, identifying and exploitation of drought-resistant genotypes, and using them as domesticated plants or involving them in correctional programs, can provide new opportunities to expand plant cultivation. *Lavandula stricta* species is native to Iran and Persian Gulf countries which are located on the dry and semi-arid belt of the world, and still no correctional program has done on this genotype. This genotype can be considered as a promising genotype to be used in correctional programs.

## Supplementary Information

Supplementary Information.

## Abbreviations

EO: Essential oil
APX: Ascorbate peroxidase
POX: Peroxidase
ROS: Reactive oxygen species
SOD: Superoxide dismutase
CAT: Catalase
MDA: Malondialdehyde
LRWC: Leaf relative water content
DPPH: 1,1-Diphenyl-2-picrylhydrazyl
H: *Lavandula angustifolia* Cv. Hidcote
M: *Lavandula angustifolia* Cv. Munstead
S: *Lavandula stricta*
I_1_: 100–90% Of field capacity (control)-irrigation regime 1
I_2_: 80–70% Of field capacity-irrigation regime 2
I_3_: 60–50% Of field capacity-irrigation regime 3
I_4_: 30–40% Of field capacity-irrigation regime 4
