# Supplementary material for: Physiological response and secondary metabolites of three lavender genotypes under water deficit
Source: Sci Rep. 2021 Sep 27;11:19164. doi: 10.1038/s41598-021-98750-x (PMC8476503; doi:10.1038/s41598-021-98750-x)
Supplement: Supplementary file 1 — Supplementary Information. [file 41598_2021_98750_MOESM1_ESM.docx]

**Supplementary Information**


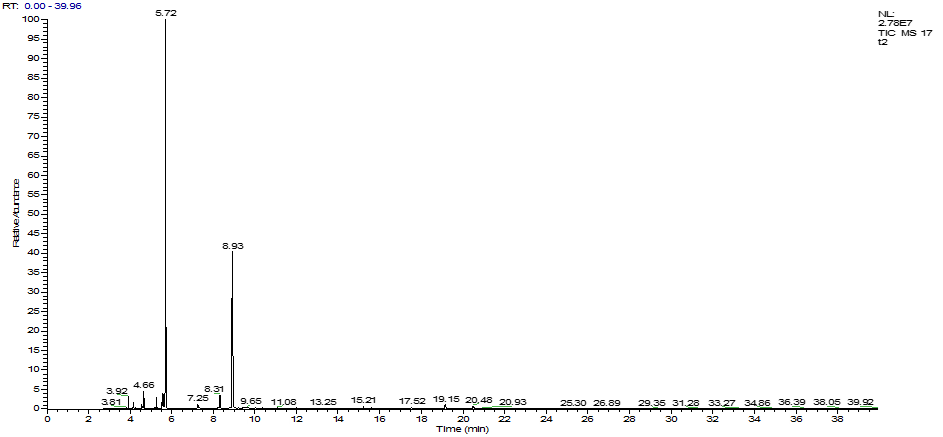


TIC chromatogram from GCMS device for *Lavandula angustifolia* cv. Hidcote under control condition (90-100% field capacity)

TIC chromatogram from GCMS device for *Lavandula stricta* under control condition (90-100% field capacity)


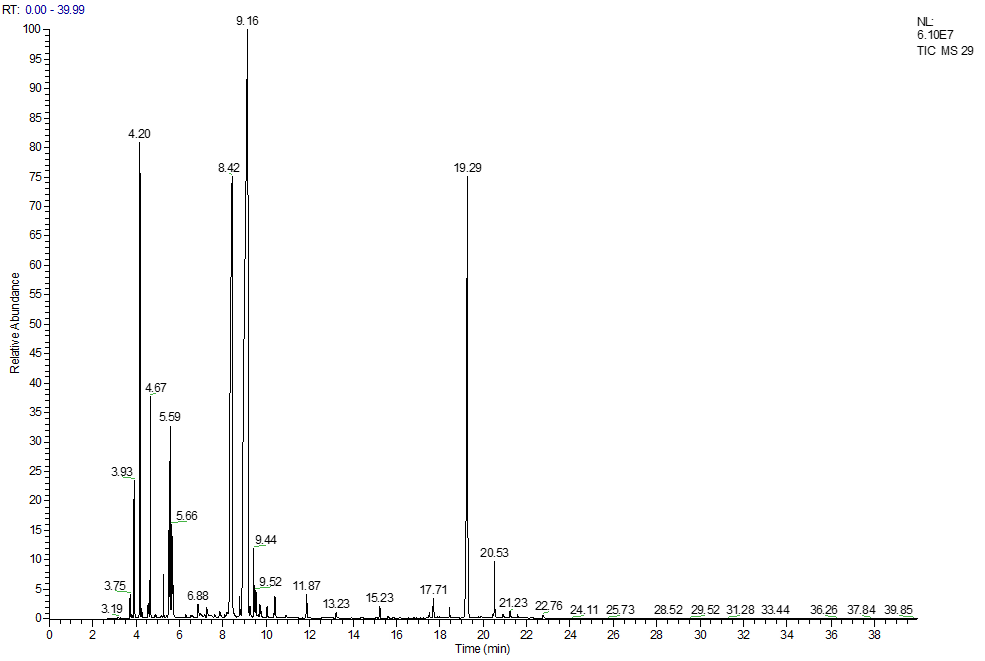


TIC chromatogram from GCMS device for *Lavandula angustifolia* cv. Munstead under control condition (90-100% field capacity)
